# Two Different Species of *Mycoplasma* Endosymbionts Can Influence Trichomonas vaginalis Pathophysiology

**DOI:** 10.1128/mbio.00918-22

**Published:** 2022-05-24

**Authors:** Valentina Margarita, Nicholas P. Bailey, Paola Rappelli, Nicia Diaz, Daniele Dessì, Jennifer M. Fettweis, Robert P. Hirt, Pier Luigi Fiori

**Affiliations:** a Department of Biomedical Sciences, University of Sassari, Sassari, Italy; b Biosciences Institute, Faculty of Medical Sciences, Newcastle University, Newcastle upon Tyne, United Kingdom; c Mediterranean Center for Disease Control (MCDC), Sassari, Italy; d Department of Microbiology and Immunology, School of Medicine, Virginia Commonwealth University, Richmond, Virginia, USA; e Department of Obstetrics and Gynecology, School of Medicine, Virginia Commonwealth University, Richmond, Virginia, USA; f Center for Microbiome Engineering and Data Analysis, Virginia Commonwealth University, Richmond, Virginia, USA; University of California, Los Angeles

**Keywords:** *Mycoplasma girerdii*, *Mycoplasma hominis*, pathogenicity, pathogroups, symbiosis, *Trichomonas vaginalis*, gene expression

## Abstract

Trichomonas vaginalis can host the endosymbiont Mycoplasma hominis, an opportunistic pathogenic bacterium capable of modulating T. vaginalis pathobiology. Recently, a new noncultivable mycoplasma, “*Candidatus* Mycoplasma girerdii,” has been shown to be closely associated with women affected by trichomoniasis, suggesting a biological association. Although several features of “*Ca.* M. girerdii” have been investigated through genomic analysis, the nature of the potential T. vaginalis-“*Ca.* M. girerdii” consortium and its impact on the biology and pathogenesis of both microorganisms have not yet been explored. Here, we investigate the association between “*Ca.* M. girerdii” and T. vaginalis isolated from patients affected by trichomoniasis, demonstrating their intracellular localization. By using an *in vitro* model system based on single- and double-*Mycoplasma* infection of *Mycoplasma*-free isogenic T. vaginalis, we investigated the ability of the protist to establish a relationship with the bacteria and impact T. vaginalis growth. Our data indicate likely competition between M. hominis and “*Ca.* M. girerdii” while infecting trichomonad cells. Comparative dual-transcriptomics data showed major shifts in parasite gene expression in response to the presence of *Mycoplasma*, including genes associated with energy metabolism and pathogenesis. Consistent with the transcriptomics data, both parasite-mediated hemolysis and binding to host epithelial cells were significantly upregulated in the presence of either *Mycoplasma* species. Taken together, these results support a model in which this microbial association could modulate the virulence of T. vaginalis.

## INTRODUCTION

Vaginal mucosal homeostasis requires an optimal combination of beneficial bacterial species, comprising a eubiotic microbiota, as well as host factors to minimize colonization opportunities for pathogenic microbes and maximize reproductive health (1). However, a complex combination of environmental factors, human genetics underlying innate and adaptive immune responses, and host physiology and behavior (2–4) can contribute to an imbalanced, dysbiotic microbiota. Dysbiosis is characterized by a highly dynamic vaginal microbial ecosystem that tends to increase the inflammatory tone of the mucosa, with diverse pathological consequences. Microbial dysbiosis contributes to pathologies of the urogenital tract, obstetric complications, and an increased risk of sexually transmitted infections such as HIV (5–7). One of the best-recognized and most common forms of vaginal dysbiosis among women of reproductive age has been defined as bacterial vaginosis (BV) (8). New -omics technologies have recently revealed that BV can be stratified into functionally different subtypes that are not resolved by more traditional diagnostic approaches (9). Most recent vaginal microbiome studies have used taxonomic surveys of the 16S rRNA gene and have thus surveyed only the bacterial composition of the microbiota. Microbial eukaryotes, including *Candida* species and Trichomonas vaginalis, can also contribute to dysbiosis in the vaginal microbial ecosystem, leading to a boost in the inflammatory tone of the vaginal tissues (10).

T. vaginalis is the causative agent of trichomoniasis, the most common nonviral sexually transmitted infection worldwide, which annually affects ~160 million men and women aged 15 to 49 years worldwide (11). Several studies have shown that T. vaginalis interaction with dysbiotic vaginal microbiota species qualitatively and quantitatively modulates the host inflammatory response, leading to pathogenesis. T. vaginalis is able to reduce the colonization of lactobacilli, which is associated with an increase in the number of anaerobic bacteria characteristic of BV, such as Fannyhessea vaginae (previously named Atopobium vaginae) (12), Prevotella bivia, *Megasphaera* sp., *Sneathia* sp., and *Gardnerella* sp. (13). More recently, *in vitro* models of polymicrobial infection revealed a correlation between *Fannyhessea* and *Gardnerella* species, two common BV-associated bacteria, alongside an enhancement of the pathogenic capabilities of T. vaginalis (14). The combination of these microbial pathogens in the vagina significantly affects the host immune response by boosting T. vaginalis-induced proinflammatory chemokine production and synergistically affecting the integrity of tight junctions between cervicovaginal epithelial cells, which together likely contribute to a reduction in mucosal barrier function *in vivo* (15, 16). The interplay between dysbiotic bacteria and T. vaginalis was further confirmed by Hinderfeld and Simoes-Barbosa, who demonstrated that biofilm produced *in vitro* by BV-associated bacteria is able to enhance the adhesion between protist and host cells, amplifying the parasite’s cytopathic effect (14). Notably, T. vaginalis clinical isolates are able to carry Mycoplasma hominis (17), recently renamed Metamycoplasma hominis (18), an opportunistic pathogenic bacterium linked with pregnancy and postpartum complications, including spontaneous abortion, endometritis, and low birth weight (19). The interaction between T. vaginalis and M. hominis is the first endosymbiosis described between two obligate human mucosal parasites producing independent diseases in the same anatomical area (20).

The presence of *M. hominis* in T. vaginalis cells has been demonstrated in clinical isolates, with an association rate ranging from 5% to over 89% (21). Several studies have demonstrated how *M. hominis* associated with T. vaginalis influences the parasite’s physiology and the dynamics of the host-parasite-bacterium interaction (22–25). More recently, a novel *Mycoplasma* species was characterized through 16S rRNA microbial surveys and metagenomic analyses. “*Candidatus* Mycoplasma girerdii,” previously referred to as “Mnola” (26) and recently renamed “*Candidatus* Malacoplasma girerdii” (18), shows an even tighter cooccurrence with T. vaginalis than *M. hominis*. The DNA of “*Ca.* M. girerdii” was detected almost exclusively in T. vaginalis-infected patients (26, 27). In addition to this specific association in the urogenital tract, 16S rRNA genes belonging to three *Mycoplasma* species, including *M. hominis* and “*Ca.* M. girerdii,” were detected in the oral cavity of a premature neonate (28), and T. vaginalis and “*Ca*. M. girerdii” genomic DNAs (gDNAs) were also found to cooccur in a premature infant’s saliva (29). In recent studies, sequences mapping to “*Ca*. M. girerdii” have been identified in several preterm birth cohorts of the vaginal microbiome (30–32), but given the low prevalence of the organism and the relatively small sample sizes of these studies, the association of “*Ca*. M. girerdii” with premature birth has yet to be adequately assessed. Notably, T. vaginalis infections are associated with several pregnancy and postpartum complications, including low birth weight, premature rupture of membranes, and preterm delivery (33). Notably, comorbidities are increasingly recognized to have important implications for diagnostics and treatment regimens during pregnancy (34). Hence, developing an understanding of the interactions between T. vaginalis and the two strongly associated *Mycoplasma* species will be essential for unraveling their respective contributions to adverse reproductive health outcomes. An improved understanding may also aid in the development of new approaches for treatment, including through nuanced modulation of the microbiota to regain vaginal eubiosis.

“*Ca*. M. girerdii” possesses typical *Mollicutes* features, such as a small genome (~619 kb), which reflects a limited metabolic capability and, thus, obligate dependence on its host as a source of essential metabolites (18). *In silico* reconstruction of metabolic pathways suggests that “*Ca.* M. girerdii” is glycolytic, similarly to Mycoplasma genitalium, and encodes all enzymes for the utilization of glucose as an energy source (27). In contrast, “*Ca.* M. girerdii” lacks gluconeogenesis, the tricarboxylic acid (TCA) cycle (Krebs cycle), and enzymes for purine, pyrimidine, and amino acid synthesis as well as the arginine dihydrolase (ADH) pathway, with the latter being essential for *M. hominis* energy metabolism (35). Notably, the “*Ca.* M. girerdii” genome also encodes proteins homologous to known microbial virulence factors, such as collagenase, hemolysin, and endopeptidase (27). A family of 26 genes encoding BspA-like proteins, containing Treponema pallidum leucine-rich repeat (TpLRR) domains (36), was also annotated in the genome of “*Ca.* M. girerdii” (27), and a larger family of genes encoding BspA-like proteins (911 members) was previously identified in T. vaginalis (37). Since some bacterial members of this protein family can stimulate a Toll-like receptor 2 (TLR2)-mediated host immune response (38), BspAs from various microbial sources may represent a common trigger of human inflammatory responses at various mucosal surfaces.

Predicted biological features of “*Ca.* M. girerdii” have been inferred through metagenomic analyses, and one very recent report supported the presumed symbiosis between T. vaginalis and “*Ca.* M. girerdii” (39). However, there are currently no data of relevance to the potential synergistic pathobiology of both microorganisms. In the current work, we provide the first molecular and mechanistic insights into this association, demonstrating that “*Ca.* M. girerdii” establishes an endosymbiotic relationship with the protist. Moreover, we present a new *in vitro* model in which isogenic mycoplasma-free T. vaginalis is infected with either “*Ca.* M. girerdii,” *M. hominis*, or both mycoplasma species. This model system is used to investigate bacterial localization, multiplicity of infection (MOI), and the role of both *Mycoplasma* species in the modulation of T. vaginalis physiopathology.

## RESULTS

### Identification of “*Ca.* M. girerdii” and *M. hominis* and their MOIs in T. vaginalis clinical isolates.

**FIG 1:**
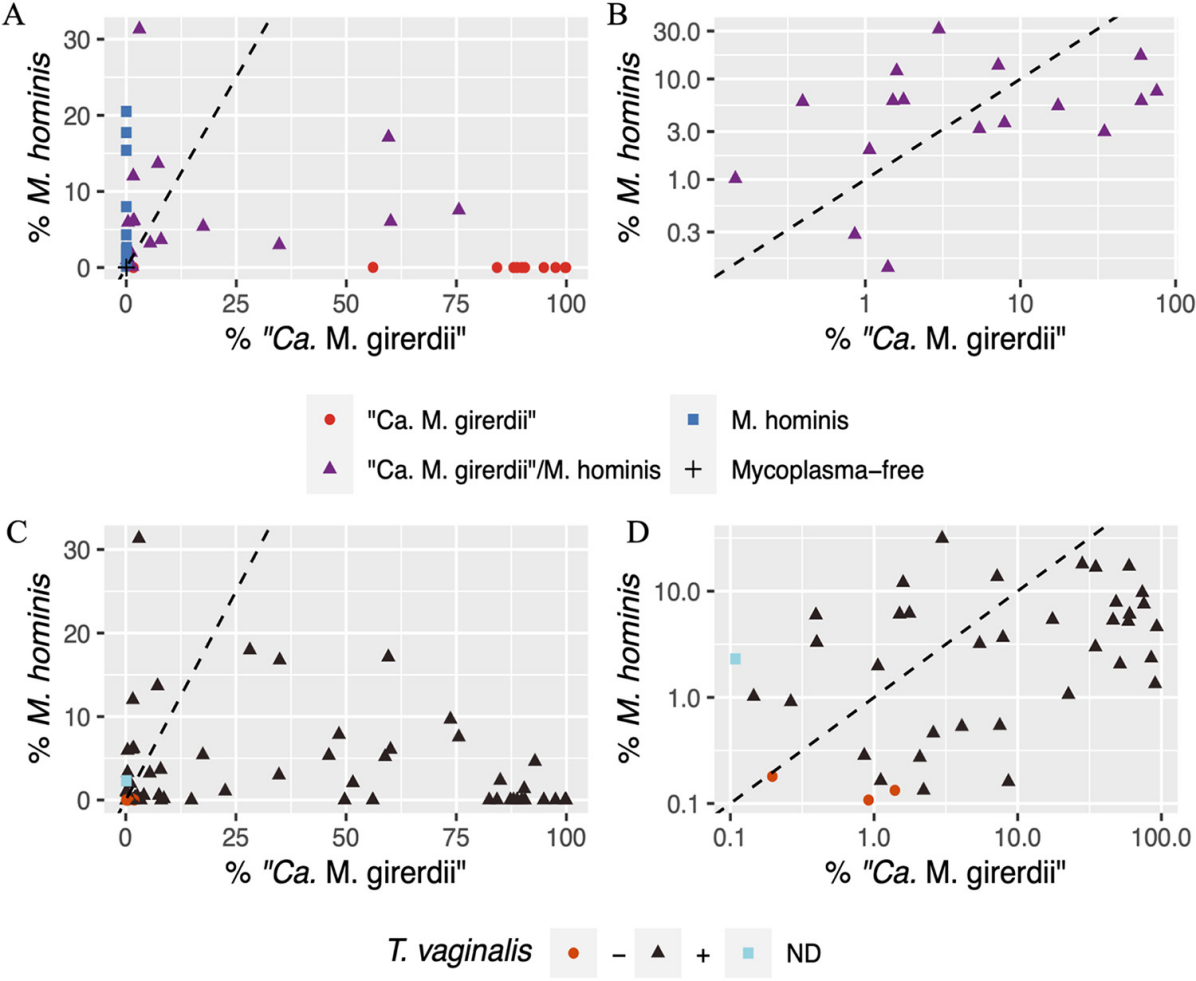
Proportion of 16S rRNA reads for “*Ca*. M. girerdii” and *M. hominis* from vaginal swabs from women either clinically diagnosed with trichomoniasis or positive for “*Ca*. M. girerdii.” (A and B) Samples from 63 women clinically diagnosed with trichomoniasis. (C and D) Samples from 73 women with at least 0.1% of 16S rRNA reads mapped to “*Ca*. M. girerdii.” ND, no data. Dashed lines indicate 1 to 1 ratio.

Analyzing the published 16S rRNA bacterial profiles of vaginal swabs (27) from 63 women diagnosed with trichomoniasis in more detail, we established that the majority (67%) of T. vaginalis-positive swabs (Real Time-PCR [RT-PCR] screening) were positive (≥0.1% 16S rRNA gene read count threshold) for either *M. hominis*, “*Ca.* M. girerdii,” or both species (Table 1 and Fig. 1A and B). Similarly, the majority (83%) of 73 women with vaginal swabs positive for “*Ca.* M. girerdii” (≥0.1% 16S rRNA gene read count threshold) were also positive for T. vaginalis (RT-PCR) (Fig. 1C and D; see also Table S1 in the supplemental material).

**TABLE 1:** Occurrence of *M. hominis* and “*Ca.* M. girerdii” among T. vaginalis isolates from vaginal swabs from trichomoniasis patients in Italy and 16S rRNA profiling of vaginal swabs from trichomoniasis patients in the United States

Isolate type	% of associated T. vaginalis strains^*a*^ (no. associated/total no. of isolates) (*n* = 75)	95% CI for no. of bacteria/trichomonad cell (mean)^*a*^	% of associated 16S rRNA profiles of women with trichomoniasis^*b*^ (no. associated/total no. of isolates) (*n* = 63)
Mycoplasma-free T. vaginalis	11 (8/75)	0	33 (21/63)
T. vaginalis infected by “*Ca*. M. girerdii”	5 (4/75)	3.7–18.24 (10.97)	17 (11/63)
T. vaginalis infected by *M. hominis*	28 (21/75)	0.0001–5.6 (2.07)	22 (14/63)
T. vaginalis infected by “*Ca*. M. girerdii” and *M. hominis*	56 (42/75)	“*Ca*. M. girerdii,” 0.003–0.1 (0.053)	27 (17/63)
		*M. hominis*, 0.001–39 (19.6)	

a Parasite isolates derived from 75 vaginal swabs from patients with acute trichomoniasis from Italy (all Caucasian) were analyzed by qPCR for the presence of *M. hominis* and “*Ca.* M. girerdii.” The percentage of strains associated with “*Ca*. M. girerdii” and/or *M. hominis* and the number of mycoplasma cells per T. vaginalis cell, evaluated by qPCR, are listed. The range and mean (in parentheses) MOI values are also listed. CI, confidence interval.

b 16S rRNA microbiome profiles from vaginal swabs from 63 patients (87.3% Black American, 4.8% Caucasian, 3.2% Hispanic/Latino, and 3.2% unknown) with acute trichomoniasis were compared for the presence of *M. hominis* and “*Ca.* M. girerdii” (threshold of ≥0.1% of total 16S rRNA mapped reads).

10.1128/mbio.00918-22.4 TABLE S1 16S rRNA profiling data from the 73 women with “*Ca*. M. girerdii.” Tv, T. vaginalis; Tv ND, T. vaginalis no data; +, positive; −, negative. A threshold of 0.1% of the total 16S rRNA reads was used as the threshold for the presence of “*Ca*. M. girerdii” and *M. hominis*. Download Table S1, DOCX file, 0.01 MB. Copyright © 2022 Margarita et al. 2022 Margarita et al. https://creativecommons.org/licenses/by/4.0/ This content is distributed under the terms of the Creative Commons Attribution 4.0 International license.

Consistent with these bacterial taxonomic surveys of clinical samples, we also identified, by quantitative real-time PCR (qPCR), one or both *Mycoplasma* species of interest among the majority (89%) of clinical isolates of T. vaginalis grown in *in vitro* cultures (Table 1). Genomic DNA from 75 T. vaginalis isolates was analyzed by qPCR performed with *M. hominis*- and “*Ca*. M. girerdii”-specific primers, demonstrating the presence of *M. hominis* DNA in 63 strains and “*Ca*. M. girerdii” DNA in 46 strains. More than half of the strains harbored both *M. hominis* and “*Ca*. M. girerdii,” whereas approximately one-third were positive for only *M. hominis* and a smaller fraction were positive for only “*Ca*. M. girerdii” (Table 1).

Using qPCR, we investigated the MOIs of “*Ca.* M. girerdii” and *M. hominis* among different T. vaginalis isolates. The number of “*Ca.* M. girerdii” bacteria per trichomonad cell was evaluated, assuming that there is a single copy of the 16S rRNA gene present per genome, as shown previously for the four sequenced strains (27). Thus, one “*Ca.* M. girerdii” 16S rRNA copy corresponded to one “*Ca*. M. girerdii” cell. Differences in DNA extraction efficiency can impact the accuracy of MOI measures (40–42). In the absence of a cell wall, “*Ca.* M. girerdii” is predicted to be as easy to lyse as other mycoplasmas and T. vaginalis. Thus, we anticipate that differences in DNA extraction efficiencies would have a minimal impact on qPCR results. The evaluation of MOIs for *M. hominis* in T. vaginalis strains was carried out assuming that one copy of the MHO_0730 gene (GenBank accession number CAX37207.1) corresponded to one *M. hominis* cell, as shown for all 17 sequenced strains labeled as “complete genomes” and with fully conserved sequences for the sites targeted by the primers.

**FIG 2:**
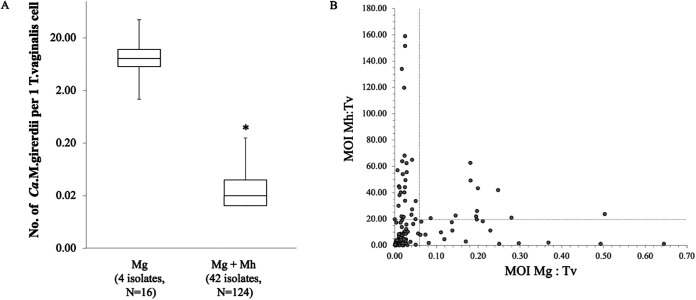
Multiplicity of infection of “*Ca*. M. girerdii” in T. vaginalis isolates. (A) Graph showing the variability in the number of bacteria per T. vaginalis cell among isolates associated exclusively with “*Ca*. M. girerdii” (Mg) and T. vaginalis strains dually infected by “*Ca*. M. girerdii” and *M. hominis* (Mg+Mh). Bars represent the means ± standard deviations (SD) from at least three independent growth experiments for each isolate of 2 isolates (Mg only) and 22 isolates (Mg+Mh), respectively. Statistical significance was tested by Student’s *t* test, and * indicates significant (*P* < 0.01) variations in terms of the number of “*Ca*. M. girerdii” bacteria between parasites associated exclusively with “*Ca*. M. girerdii” and those in symbiosis with both *M. hominis* and “*Ca*. M. girerdii.” (B) Plot area illustrating the relationship between the *M. hominis* MOI and the “*Ca*. M. girerdii” MOI in 22 dually infected T. vaginalis (Tv) strains. There are a total of 65 points corresponding to replicate cultures for the 22 dually infected strains. The negative trend observed among the 22 dual symbioses implies some form of direct competition between the two bacterial species. The horizontal dotted line indicates the mean value of the *M. hominis* MOI, while the vertical dotted line indicates the mean value of the MOI of “*Ca*. M. girerdii,” among the 22 dually infected T. vaginalis clinical isolates.

Using this approach, we observed that when “*Ca.* M. girerdii” was associated exclusively with T. vaginalis, the estimated mean MOI value was ~11 bacteria per T. vaginalis cell (Table 1). Notably, when both *M. hominis* and “*Ca.* M. girerdii” were present in the same T. vaginalis isolate, the “*Ca.* M. girerdii” MOI massively decreased from a ratio of ~11:1 to ~1:20 bacteria per T. vaginalis cell (Fig. 2A and Table 1). In contrast, the *M. hominis* MOI in symbiosis with T. vaginalis was 2:1, which increased to a mean value of ~20:1 in the context of dual symbiosis (Table 1).

These results indicate important isolate-to-isolate variability in the number of *M. hominis* and “*Ca*. M. girerdii” bacteria per T. vaginalis cell and that the presence of *M. hominis* has a significant impact on the ability of “*Ca*. M. girerdii” to grow within the parasite and vice versa. The inhibitory effect of the presence of *M. hominis* on the “*Ca*. M. girerdii” MOI among the 42 dual symbioses suggests direct competition between the bacterial species (Fig. 2B; Table S2). Furthermore, when comparing the 16S rRNA profiles of vaginal swabs (27), the proportion of reads mapping to “*Ca*. M. girerdii” or *M. hominis* 16S rRNA genes is also consistent with the two bacteria influencing each other (Fig. 1; Table S1).

10.1128/mbio.00918-22.5 TABLE S2 Number of *Mycoplasma* species associated with 22 different T. vaginalis isolates. Tv, T. vaginalis; Mg, “*Ca*. M. girerdii”; Mh, *M. hominis*. Download Table S2, DOCX file, 0.02 MB. Copyright © 2022 Margarita et al. 2022 Margarita et al. https://creativecommons.org/licenses/by/4.0/ This content is distributed under the terms of the Creative Commons Attribution 4.0 International license.

### Intracellular localization of “*Ca.* M. girerdii” in T. vaginalis cells: gentamicin protection and fluorescence assays.

**FIG 3:**
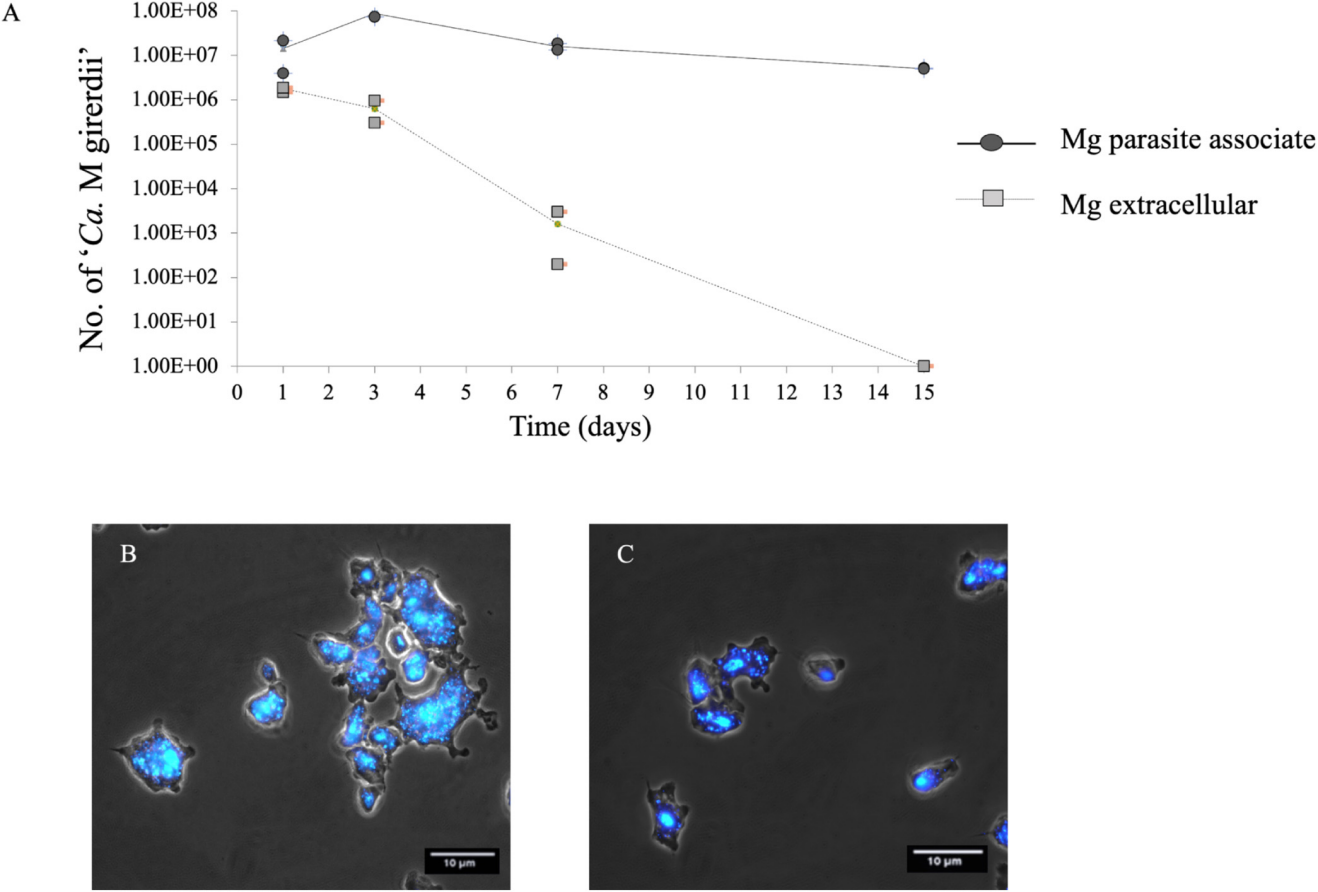
Effect of gentamicin treatment on “*Ca.* M. girerdii” associated with T. vaginalis. (A) TvSS-62Mg, a naturally “*Ca*. M. girerdii”-infected T. vaginalis isolate, was grown for 2 weeks in medium complemented with gentamicin in order to evaluate the susceptibility of the bacteria to the antibiotic treatment. Two replicate experiments were performed, and both data points are indicated. DNA from 1.00E+06 T. vaginalis cells/mL was extracted and analyzed by qPCR after 1, 3, 7, and 15 days of incubation with gentamicin. The curves refer to the number of “*Ca*. M. girerdii” bacteria found in association with the parasite (Mg parasite associate) and in the supernatant of cell cultures (extracellular) treated with gentamicin. The presence of live bacteria associated with T. vaginalis after up to 15 days of antibiotic treatment and their eventual disappearance after 15 days in the supernatant indicate that “*Ca*. M. girerdii” bacteria were able to multiply intracellularly, protected from the antibiotic. The intracellular persistence of “*Ca*. M. girerdii” in trichomonad cells after 15 days of gentamicin treatment was further supported by fluorescence (see panel C). (B) DAPI staining of TvSS-62Mg in the absence of exposure to gentamicin. (C) DAPI staining obtained following 2 weeks of antibiotic treatment of TvSS-62Mg cultures, indicating the presence of intracellular bacteria and consistent with the qPCR data illustrated in panel A.

T. vaginalis strain SS-62 (TvSS-62Mg) was treated with gentamicin at a bactericidal concentration of 50 μg mL^−1^ in order to investigate whether “*Ca.* M. girerdii” is able to survive in the trichomonad cytoplasm, as the antibiotic does not enter the parasite (43–45). The susceptibility of “*Ca.* M. girerdii” to gentamicin was confirmed by the inability of gentamicin-treated supernatants to infect mycoplasma-free parasites. Aliquots were collected at days 1, 3, 7, and 15 during the gentamicin protection assay; subjected to total DNA extraction; and analyzed by qPCR to detect intracellular (T. vaginalis pellet) and extracellular (supernatant) “*Ca.* M. girerdii” DNA. As shown in Fig. 3A, “*Ca*. M. girerdii” DNA was detected in both trichomonad cells and the supernatant after up to 1 week of gentamicin treatment. Notably, after 15 days of cultivation in medium with gentamicin, “*Ca*. M. girerdii” DNA was still detected in T. vaginalis cells, while in contrast, it could not be detected in the corresponding supernatants of antibiotic-exposed cultures. The presence of bacteria in the parasite pellet demonstrates that they were able to survive the antibiotic treatment, thus suggesting an intracellular localization.

In order to further investigate the presence of “*Ca*. M. girerdii” in TvSS-62Mg after 2 weeks of cultivation in medium complemented with gentamicin, we performed a fluorescence assay. Mycoplasma-associated parasites were stained using 4′,6-diamidino-2-phenylindole (DAPI) and analyzed by fluorescence microscopy. Figure 3B shows the clear presence of “*Ca.* M. girerdii” in the control T. vaginalis culture before treatment with the antibiotic, confirming the association between the parasite and mycoplasma under these *in vitro* culture conditions. Notably, Fig. 3C illustrates T. vaginalis cells still hosting intracellular “*Ca.* M. girerdii” after 15 days of cultivation in the presence of gentamicin, consistent with intracellular bacterial growth.

### Ability of “*Ca.* M. girerdii” to infect T. vaginalis strains in the presence or absence of *M. hominis*.

**FIG 4:**
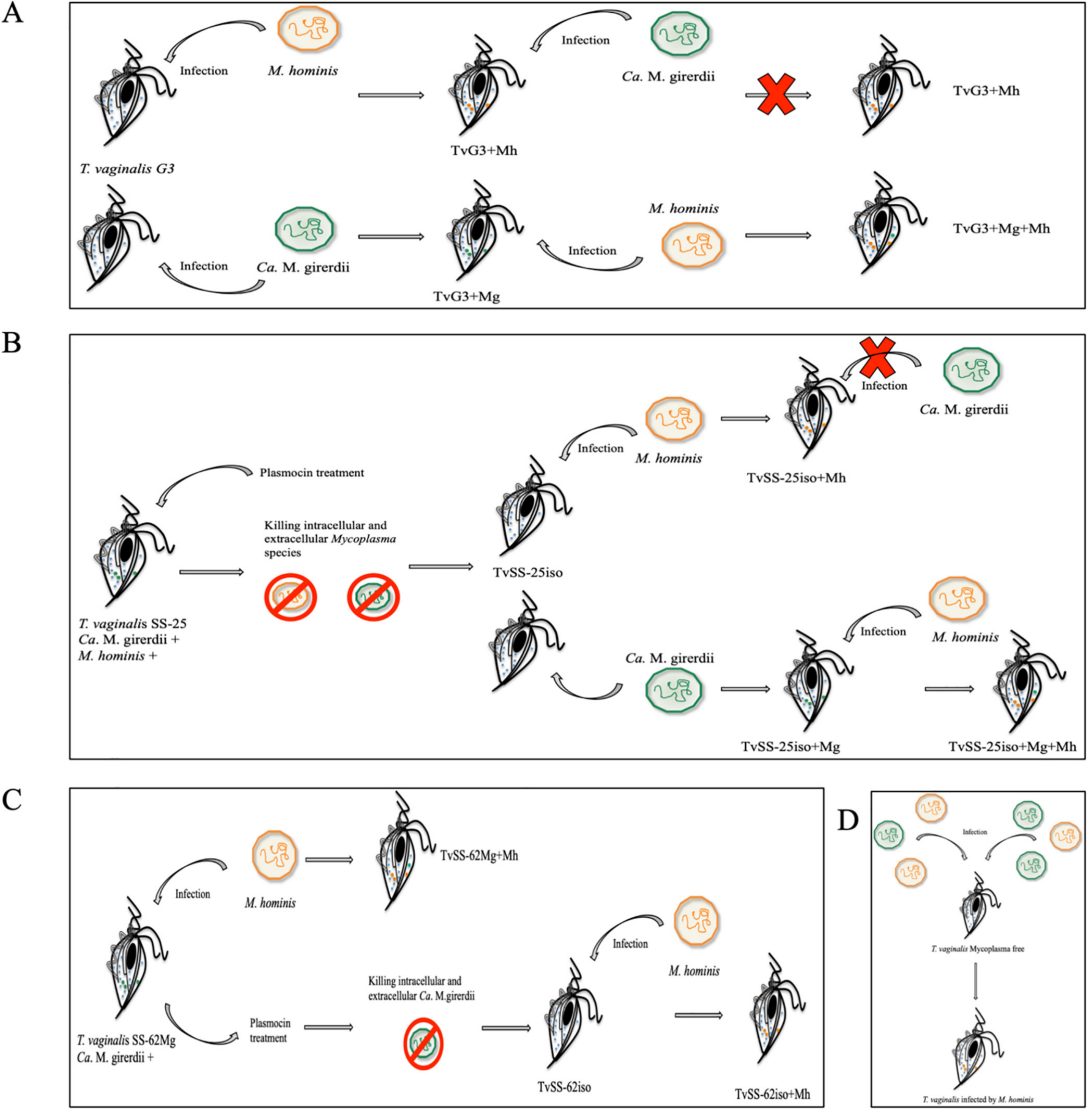
*In vitro* model system developed to study the ability of *Mycoplasma* to infect T. vaginalis cells. Three T. vaginalis isolates were used to evaluate the capability of infection of *Mycoplasma* species: T. vaginalis strain G3 (TvG3), mycoplasma free; T. vaginalis SS-25 (TvSS-25MgMh), naturally infected with “*Ca.* M. girerdii” and *M. hominis*; and T. vaginalis SS-62 (TvSS-62Mg), naturally “*Ca.* M. girerdii” infected. (A) Approach used to infect T. vaginalis strain G3 with *M. hominis* and “*Ca.* M. girerdii.” (B and C) Experimental approaches used to clear mycoplasma species from T. vaginalis MgMh (B) and T. vaginalis SS-62Mg (C) to obtain mycoplasma-free cultures (TvSS-25iso and TvSS-62iso) that were subsequently infected with *M. hominis* (TvSS-25iso+Mh and TvSS-62iso+Mh) and “*Ca.* M. girerdii” (TvSS-25iso+Mg). (D) When *M. hominis* and “*Ca.* M. girerdii” were added at the same time to *Mycoplasma*-free T. vaginalis cultures, only *M. hominis* was able to establish a stable infection with *Trichomonas* cells.

The ability of “*Ca.* M. girerdii” to establish a stable symbiotic relationship among T. vaginalis isolates in the presence or absence of *M. hominis* was studied *in vitro* using different parasite strains as recipients. In the first group of experiments, we coincubated the mycoplasma-free T. vaginalis reference strain G3 (TvG3) (46) with “*Ca.* M. girerdii” by using the same experimental strategy as the one described previously to infect T. vaginalis with *M. hominis* (24). The filtered supernatant of T. vaginalis isolate TvSS-62Mg, containing an average of 2.07E+06 “*Ca.* M. girerdii” bacteria, was added daily to a mid-log-phase culture of TvG3. The symbiosis between TvG3 and “*Ca.* M. girerdii” was confirmed by qPCR, showing the ability of this mycoplasma species to invade the parasite host (Fig. 4A). However, we noted that after ~10 freeze-thaw cycles during the storage of TvG3 in symbiosis with “*Ca.* M. girerdii” in liquid nitrogen, or following cultivation over longer periods (daily passages over 2 months), TvG3 could not maintain a stable association with “*Ca.* M. girerdii.” In contrast, a number of T. vaginalis strains, including TvG3, are able to maintain a stable symbiotic relationship over time with *M. hominis* (47). These data suggest that when a mycoplasma-free T. vaginalis strain is used as a recipient, the *in vitro* symbiosis with “*Ca*. M. girerdii” is less stable than with *M. hominis*.

Due to the instability of the symbiosis between TvG3 and “*Ca.* M. girerdii,” we studied the mycoplasma-parasite relationship using a different experimental approach. Two T. vaginalis strains, TvSS-25MgMh and TvSS-62Mg, naturally infected either by both *M. hominis* and “*Ca.* M. girerdii” or by “*Ca.* M. girerdii,” respectively, were treated with Plasmocin (48), resulting in the corresponding axenic mycoplasma-free strains (TvSS-25iso and TvSS-62iso). TvSS-62iso and T. vaginalis strain G3, which is naturally *Mycoplasma* free, were cultivated for 30 days to evaluate the influence of treatment on trichomonad growth. As shown in Fig. S1A, the growth of TvSS-62iso is not significantly distinct from the growth of TvG3. Subsequently, TvSS-25iso and TvSS-62iso were used as recipients in order to produce *in vitro* single- and double-mycoplasma infections (Fig. 4B and C). This experimental model showed that “*Ca.* M. girerdii” is able to form a stable symbiosis with both trichomonad strains cultivated with daily passages over 15 days, suggesting that a previous adaptation to host mycoplasma, either *M. hominis* or “*Ca.* M. girerdii,” can predispose a subsequent stable infection by “*Ca.* M. girerdii” under the conditions of these experiments. Notably, both T. vaginalis strains, when previously infected with “*Ca.* M. girerdii,” can form a symbiosis with *M. hominis* but not vice versa, suggesting that symbiosis with *M. hominis* inhibits subsequent symbiosis with “*Ca.* M. girerdii,” while the presence of “*Ca.* M. girerdii” does not block symbiosis with *M. hominis* under the tested conditions (Table 2).

**TABLE 2:** T. vaginalis strains used as recipients to produce isogenic trichomonad strains with single and double infections

Recipient strain^*a*^	No. of bacteria used to infect T. vaginalis^*b*^	Isogenic strain obtained^*c*^	95% CI of no. of bacteria/trichomonad cell (mean)^*d*^
TvG3	2.07E+06 of “*Ca*. M. girerdii”	TvG3+Mg	“*Ca*. M. girerdii,” 2.66–5.08 (3.9)
TvG3	1.09E+05 of *M. hominis*	TvG3+Mh	*M. hominis*, 0.03–0.45 (0.24)
TvG3+Mg	3.88E+04 of *M. hominis*	TvG3+Mg+Mh	“*Ca*. M. girerdii,” 0.13–0.27 (0.2)
			*M. hominis*, 0.004–0.01 (0.007)
TvSS-25iso	2.16E+06 of “*Ca*. M. girerdii”	TvSS-25iso+Mg	“*Ca*. M. girerdii,” 0.24–0.42 (0.33)
TvSS-25iso	5.25E+03 of *M. hominis*	TvSS-25iso+Mh	*M. hominis*, 1.31–1.34 (1.33)
TvSS-25iso+Mg	3.60E+05 of *M. hominis*	TvSS-25iso+Mg+Mh	“*Ca*. M. girerdii,” 0.005
			*M. hominis*, 1.1–1.53 (1.3)
TvSS-62iso	1.50E+06 of *M. hominis*	TvSS-62iso+Mh	*M. hominis*, 0.82–1.1 (0.95)
TvSS-62Mg	3.60E+05 of *M. hominis*	TvSS-62Mg+Mh	“*Ca*. M. girerdii,” 0.17–0.42 (0.3)
			*M. hominis*, 0.08–0.14 (0.11)

a T. vaginalis G3 is naturally mycoplasma free, T. vaginalis SS-25iso (TvSS-25iso) and T. vaginalis SS-62iso (TvSS-62iso) are strains experimentally cleaned from *Mycoplasma* species, and T. vaginalis SS-62 (TvSS-62Mg) is naturally “*Ca*. M. girerdii” infected (mean MOI, 11.83).

b Number of bacteria used to infect the recipient strains evaluated by qPCR.

c Isogenic strains experimentally obtained after infection with the indicated *Mycoplasma* species.

d Ranges and means (in parentheses) of MOI values for each T. vaginalis strain experimentally infected after 15 days of culture (continuous passage every day) are shown. The number of strains tested to evaluate the MOI of bacteria was 3 under each condition.

10.1128/mbio.00918-22.1 FIG S1 Dynamics of infection TvSS-62 isogenic strains. (A) T. vaginalis experimentally cleaned from “*Ca*. M. girerdii” and T. vaginalis G3, naturally mycoplasma free, were cultivated for 30 days in order to assess the influence of Plasmocin treatment on the growth of the protist. (B) Numbers of “*Ca*. M. girerdii” bacteria were compared between T. vaginalis SS-62, naturally “*Ca*. M. girerdii” infected, and TvSS-62Mg, experimentally infected with *M. hominis*. As shown in the graph, the number of “*Ca*. M. girerdii” bacteria statistically decreases when *M. hominis* infection is stabilized in trichomonad cells (*P* < 0.01). Download FIG S1, DOCX file, 0.1 MB. Copyright © 2022 Margarita et al. 2022 Margarita et al. https://creativecommons.org/licenses/by/4.0/ This content is distributed under the terms of the Creative Commons Attribution 4.0 International license.

The kinetics of infection obtained by comparing the MOI values of *Mycoplasma* in TvSS-62 and TvSS-62Mg+Mh confirmed the data from the clinical isolates: in the presence of a stable *M. hominis* infection, the number of “*Ca.* M. girerdii” bacteria associated with T. vaginalis decreases, compared with T. vaginalis associated with “*Ca.* M. girerdii” only (Fig. S1B).

**FIG 5:**
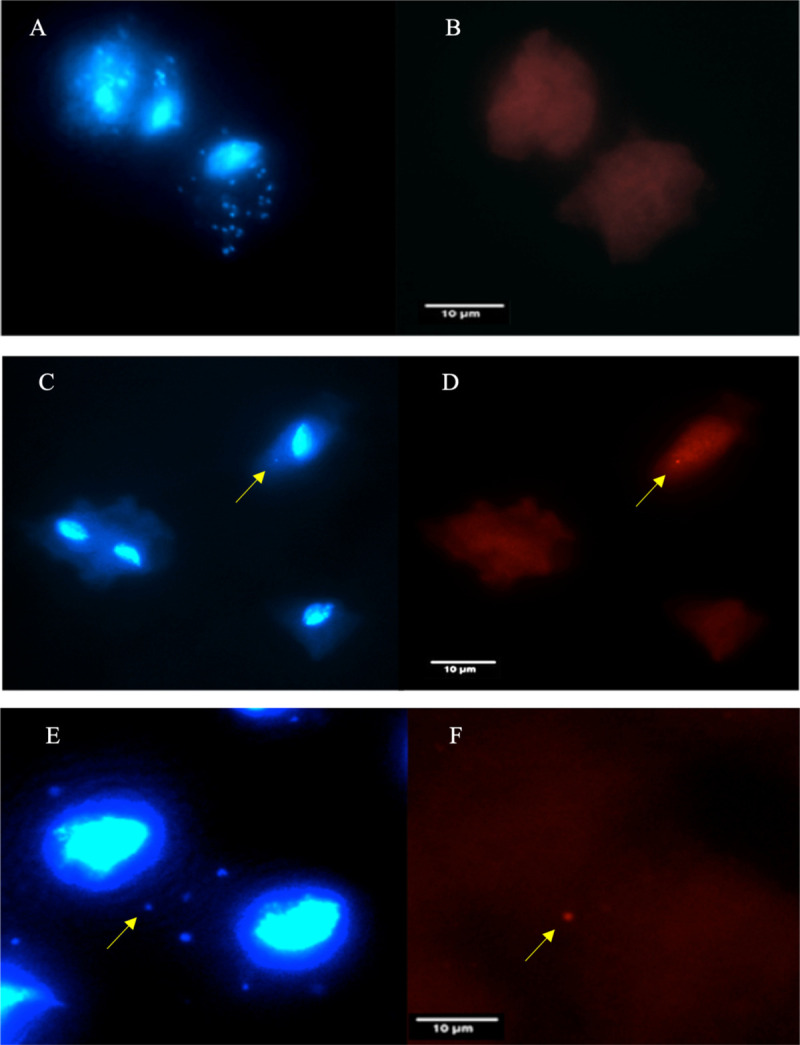
Detection of *Mycoplasma* species in T. vaginalis cells. (A) Cellular localizations of “*Ca*. M. girerdii” in TvSS-62Mg and a high number of bacteria stained with DAPI (22 stained cells). (B) The absence of *M. hominis* infection was demonstrated by using anti-*M. hominis* antibodies. (C and D) The presence of a very low number of *M. hominis* bacteria in T. vaginalis experimentally infected with the bacterium (TvSS-62iso+Mh), with just one stained cell in this one T. vaginalis cell. Yellow arrows indicate the localization of *M. hominis* in a trichomonad cell stained with DAPI (C) and with anti-*M. hominis* antibody (D). (E and F) Localization of “*Ca*. M. girerdii” and *M. hominis* stained with DAPI (E) and with anti-*M. hominis* antibody (F) in T. vaginalis SS-62Mg+Mh cells. Yellow arrows indicate the localization of *M. hominis* in a trichomonad cell stained with DAPI (C and E) and with anti-*M. hominis* antibody (D and F), with the other DAPI-labeled cells representing “*Ca*. M. girerdii” cells.

The qPCR-based quantification data were further supported by a fluorescence assay. TvSS-62Mg can host a high number of bacteria (Fig. 5A) (mean MOI of 15:1). The absence of *M. hominis* in TvSS-62Mg was also confirmed by the absence of bacteria labeled by anti-*M. hominis* antibodies (Fig. 5B).

The presence of *M. hominis* in TvSS-62iso+Mh was confirmed by combining DAPI (Fig. 5C) and staining using anti-*M. hominis* antibodies (Fig. 5D) after 15 days of symbiosis, demonstrating the association of bacteria with trichomonad cells over the tested time frame. The low number of intracellular *M. hominis* bacteria was confirmed via the qPCR results (mean MOI of 0.8:1).

The localization of both mycoplasma species in TvSS-62Mg+Mh experimentally exposed to *M. hominis* is illustrated in Fig. 5E and F. The presence of *M. hominis* was demonstrated by using specific antibodies. As previously demonstrated by the qPCR-based quantifications, the MOI of “*Ca*. M. girerdii” in parasites experimentally coinfected with *M. hominis* is significantly lower (Table 2) (TvG3+Mg+Mh, MOI of 0.13 to 0.27 [mean, 0.2]; TvSS-25iso+Mg+Mh, mean MOI of 0.0005; TvSS-62Mg+Mh, MOI of 0.17 to 0.42 [mean, 0.3]) than that observed in T. vaginalis naturally infected with “*Ca.* M. girerdii” prior to coinfection (Table 1) (MOI, 3.7 to 18.24 [mean MOI of 11]) (Fig. 5A).

### Effects of mycoplasma species on the growth rate of T. vaginalis cultures.

**FIG 6:**
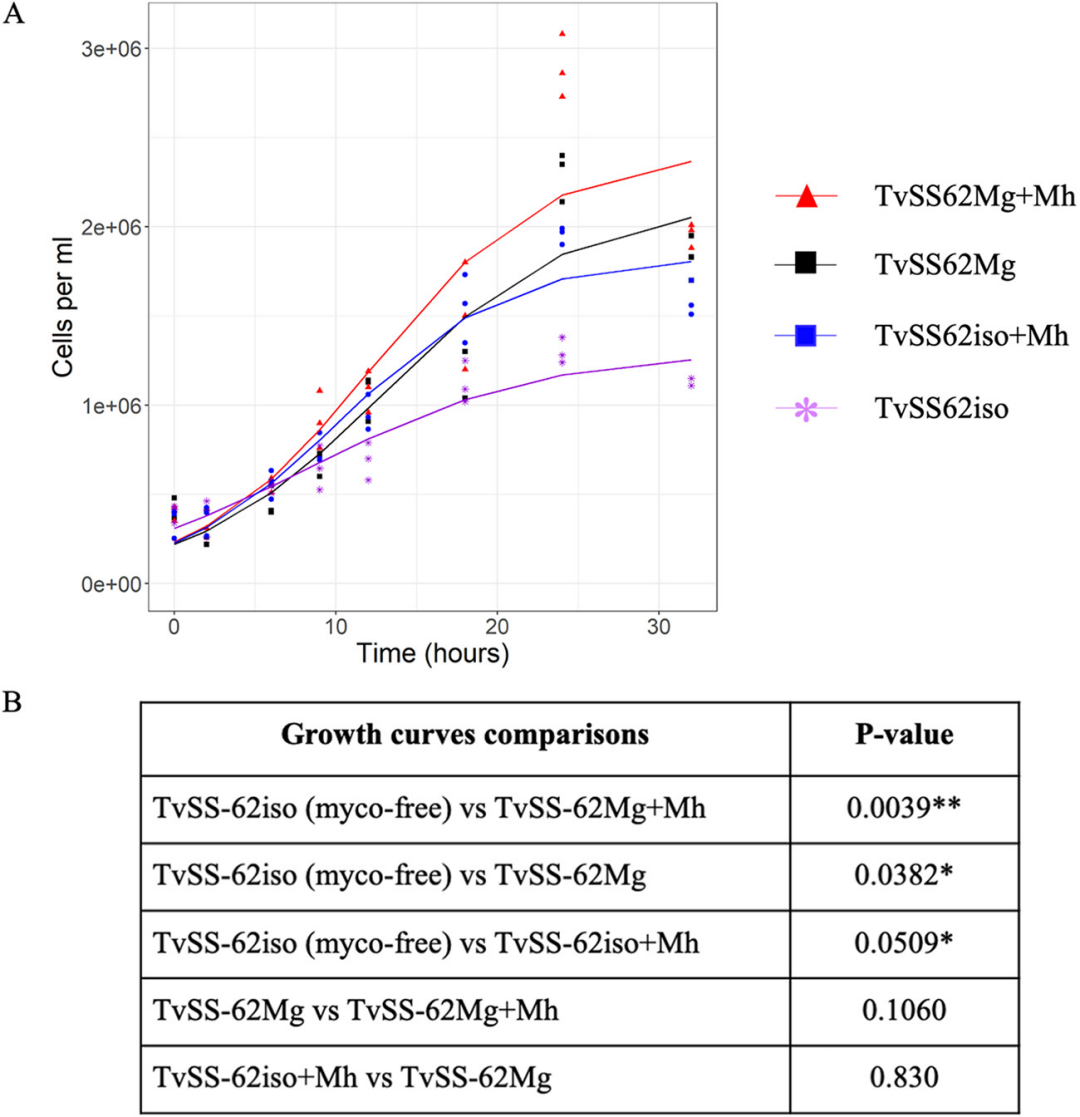
Influence of *Mycoplasma* species on T. vaginalis growth curves. Under all conditions, 400,000 parasite cells/mL were used as the starting point for the growth experiments, and data were collected at the indicated times. The growth curves are characterized by 3 parameters (*K*, *N*_0_, and *r*, respectively [see the equation as a function of the growth rate in Materials and Methods]) for each of the following strains: TvSS-62iso (1,301,849, 309,953, and 0.139), TvSS-62iso+Mh (1,834,817, 228,428, and 0.189), TvSS-62Mg (2,137,960, 220,307, and 0.167), and TvSS-62Mg+Mh (2,430,753, 232,721, and 0.183). The rates of replication of isogenic mycoplasma-free (myco-free) T. vaginalis TvSS-62iso (purple), *M. hominis*-infected T. vaginalis TvSS-62iso+Mh (blue), naturally “*Ca*. M. girerdii”-infected T. vaginalis TvSS-62Mg (black), and *M. hominis*- and “*Ca*. M. girerdii”-infected T. vaginalis TvSS-62Mg+Mh (red) were compared (A), and the presence of a single *Mycoplasma* species or both bacteria in trichomonad cells was associated with increased growth rates of the parasites, compared with the replication rate of mycoplasma-free T. vaginalis (*, *P* < 0.05; **, *P* < 0.01) (B).

We compared the growth of TvSS-62Mg, naturally infected with “*Ca.* M. girerdii,” with that of the isogenic mycoplasma-free T. vaginalis strain (TvSS-62iso). In the same experiment, we also evaluated the growth rates of *M. hominis*-infected T. vaginalis (TvSS-62iso+Mh) and dually *M. hominis*- and “*Ca.* M. girerdii”-infected T. vaginalis (TvSS-62Mg+Mh). The parasites, in various associations with bacteria, were cultured for a total of 36 h, and total DNA was extracted from the parasites to quantify “*Ca.* M. girerdii” and *M. hominis* DNAs by qPCR. The variation between the growth curves of the mycoplasma-free T. vaginalis isogenic strain (TvSS-62iso) and the T. vaginalis doubly infected strain (TvSS-62Mg+Mh) was significant (*P* value of <0.01), with a higher rate of replication for TvSS-62Mg+Mh than for TvSS-62iso. There was also significant variation (*P* value of <0.05) between the growth curves of the mycoplasma-free T. vaginalis strain (TvSS-62iso) and both T. vaginalis infected by “*Ca*. M. girerdii” alone (TvSS-62Mg) and T. vaginalis infected by *M. hominis* alone (TvSS-62iso+Mh), with higher replication rates for TvSS-62Mg and TvSS-62iso+Mh than for TvSS-62iso. In contrast, the differences between TvSS-62Mg and both TvSS-62iso+Mh and TvSS-62Mg+Mh were not statistically significant (Fig. 6).

These results indicate that both “*Ca*. M. girerdii” and *M. hominis*, in single or double infections, are associated with an increased rate of replication of T. vaginalis in Diamond’s Trypticase-yeast extract-maltose (TYM) medium.

### RNA sequencing (RNA-Seq) analysis of T. vaginalis associated with *Mycoplasma*.

**FIG 7:**
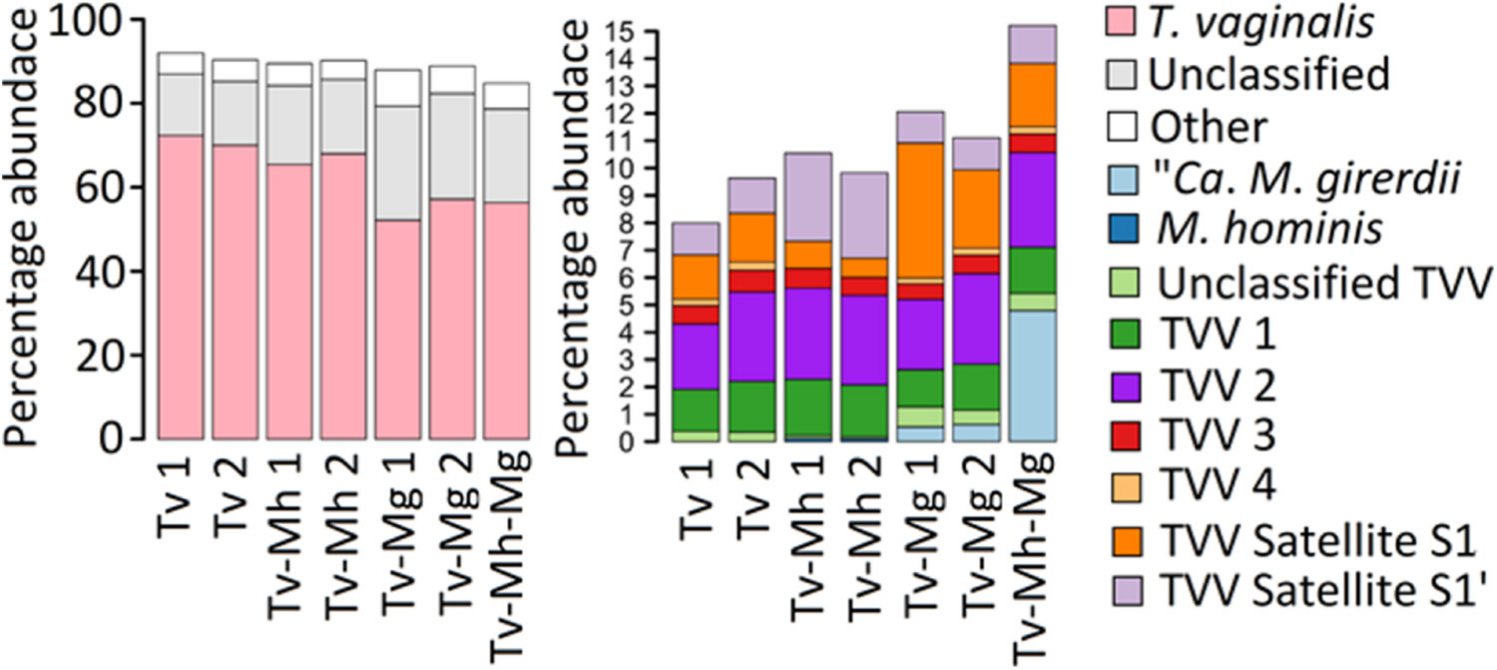
Number of reads allocated by Kraken2 to each organism under the different culture conditions. The most abundant groups (A) and less abundant groups (B) are shown separately as a percentage of the library size (TVV, Trichomonas vaginalis virus). “Unclassified” reads could not be aligned by Kraken2 to any sequence in the NCBI nonredundant nucleotide database.

Reads from TvSS-62Mg, TvSS-62iso+Mh, TvSS-62Mg+Mh, and TvSS-62iso (axenically cleaned from “*Ca*. M. girerdii” and used as a control) were classified by Kraken2 (Fig. 7). T. vaginalis reads were the most abundant in all libraries, ranging from 53 to 73% of the total reads, as expected. Consistent with the relatively lower MOI for *M. hominis* TvSS-62iso+Mh (MOI range, 0.82 to 1.1 [mean, 0.95]; *n* = 3), the reads mapping onto the reference genome of *M. hominis* had relatively lower abundances in the TvSS-62iso+Mh sample (0.11% of the total reads), whereas “*Ca.* M. girerdii” reads were more abundant in TvSS-62Mg (0.57%), suggesting a lower overall biomass of *M. hominis* (Fig. 7B). *M. hominis* reads decreased by a factor of nearly 100 in TvSS-62Mg+Mh (0.0016%) compared with TvSS-62iso+Mh symbiosis, whereas “*Ca.* M. girerdii” reads were more frequent in TvSS-62Mg (4.7% of the total reads). Evidence from read classification indicated the presence of several distinct Trichomonas vaginalis viruses (TVVs) and associated satellite viruses. Viral reads were relatively abundant, ranging from 7.7 to 11% of the total reads. Intriguingly, the presence of *Mycoplasma* appeared to have an influence on the relative abundance of some viral transcripts. Compared with the other conditions, TVV4 reads were greatly decreased in T. vaginalis associated with *M. hominis* only (TvSS-62iso+Mh). TVV satellite S1 reads were more abundant in the TvSS-62Mg strain, and TVV satellite S1′ appeared to be enhanced by the presence of *M. hominis* alone.

**FIG 8:**
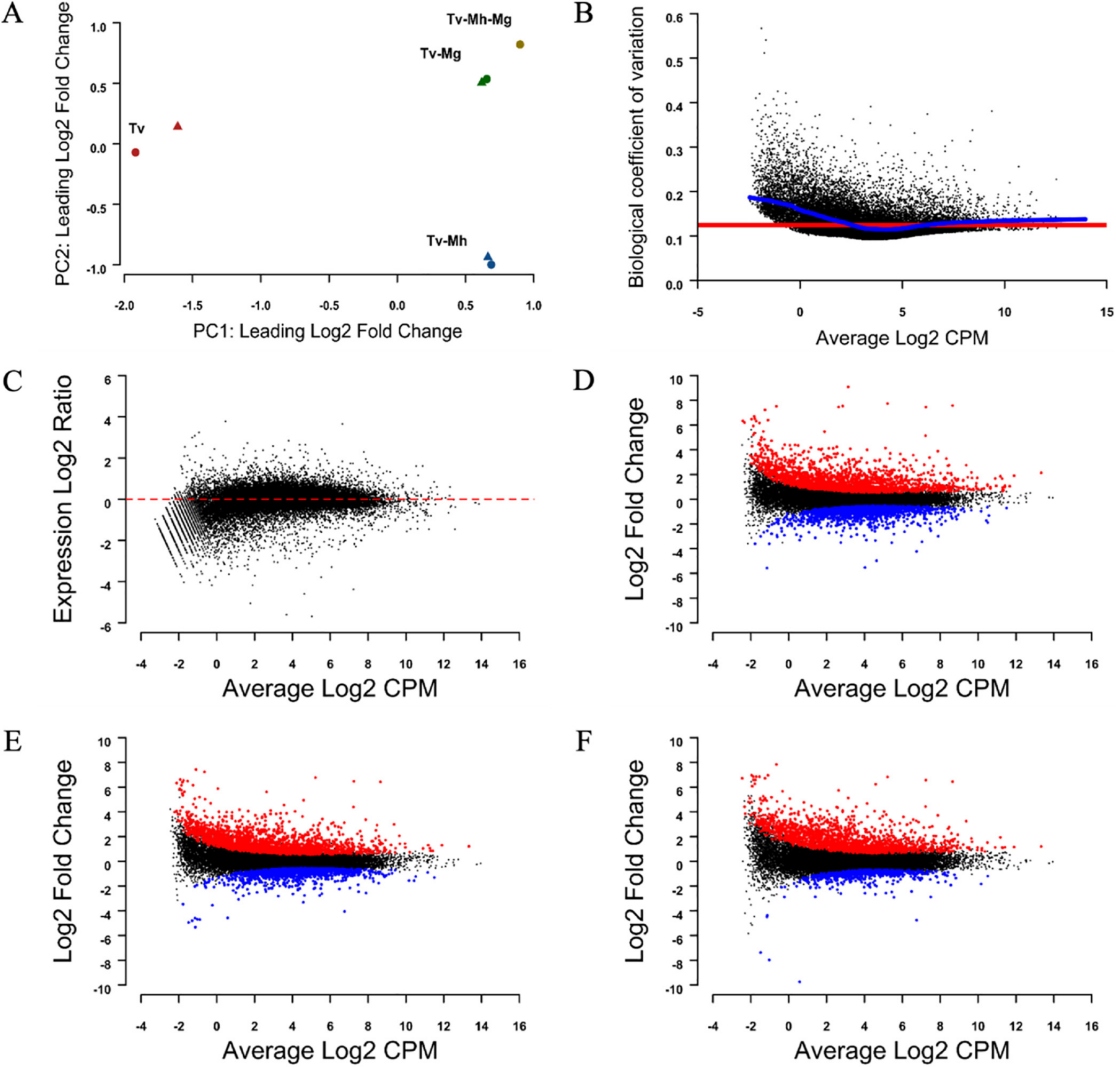
Quality assessment for RNA-Seq expression quantification data. (A) Principal-coordinate analysis (PCoA) showing root mean square deviations of log_2_ fold changes for all genes between samples. Samples 1 and 2 under each condition are indicated by circles and triangles, respectively. (B) Biological coefficient of variation (BCV) versus log_2_ counts (counts per million [CPM]) for each gene throughout all samples. The trended and common BCV estimates are shown in blue and red, respectively. (C) Smear plot showing log_2_ expression ratios for a single T. vaginalis (TvSS-62iso) sample compared with the average for all others versus log_2_ read counts. The dashed line indicates zero. (D to F) Smear plots showing log_2_ fold changes compared with the control (T. vaginalis [Tv]) for TvSS-62iso+Mh (Tv-Mh) (D), TvSS-62Mg (Tv-Mg) (E), and TvSS-62Mg+Mh (Tv-Mh-Mg) (F) versus average expression, with significantly upregulated and downregulated genes (FDR < 0.05) highlighted in red and blue, respectively.

Assessment of the quantification data mapped onto the T. vaginalis G3 reference genome suggested that the data are of high quality and suitable for assessing differential expression. Principal-coordinate analysis (PCA) demonstrated that the variation between conditions was much greater than that between replicates. PCA also suggested greater similarity within the *Mycoplasma*-infected samples than with *Mycoplasma*-free T. vaginalis (TvSS-62iso) and greater similarity between TvSS-62Mg and TvSS-62Mg+Mh conditions than for the TvSS-62iso+Mh condition (Fig. 8A). The biological coefficient of variation (BCV) among the data was low (common estimate across all genes, 0.015) (Fig. 8B), and the majority of genes showed low variation of expression between samples after statistical normalization (Fig. 8C), indicating that normalization was successful. A total of 5,938 genes were significantly differentially expressed across all *Mycoplasma* conditions compared with the control TvSS-62iso (Table S3). *M. hominis* alone induced the largest response, with 3,814 significantly modulated genes, followed by 3,617 in response to *M. girerdii* and, intriguingly, only 2,558 in response to simultaneous *M. hominis* and *M. girerdii* infection (Fig. 8D to F; Table S3).

10.1128/mbio.00918-22.6 TABLE S3 Output DE genes. Data from RNA-Seq analysis of T. vaginalis associated with *Mycoplasma* are shown (https://figshare.com/s/dbd42153e61a46803187). Download Table S3, DOCX file, 0.01 MB. Copyright © 2022 Margarita et al. 2022 Margarita et al. https://creativecommons.org/licenses/by/4.0/ This content is distributed under the terms of the Creative Commons Attribution 4.0 International license.

### T. vaginalis genes regulated in the presence of *Mycoplasma*.

**FIG 9:**
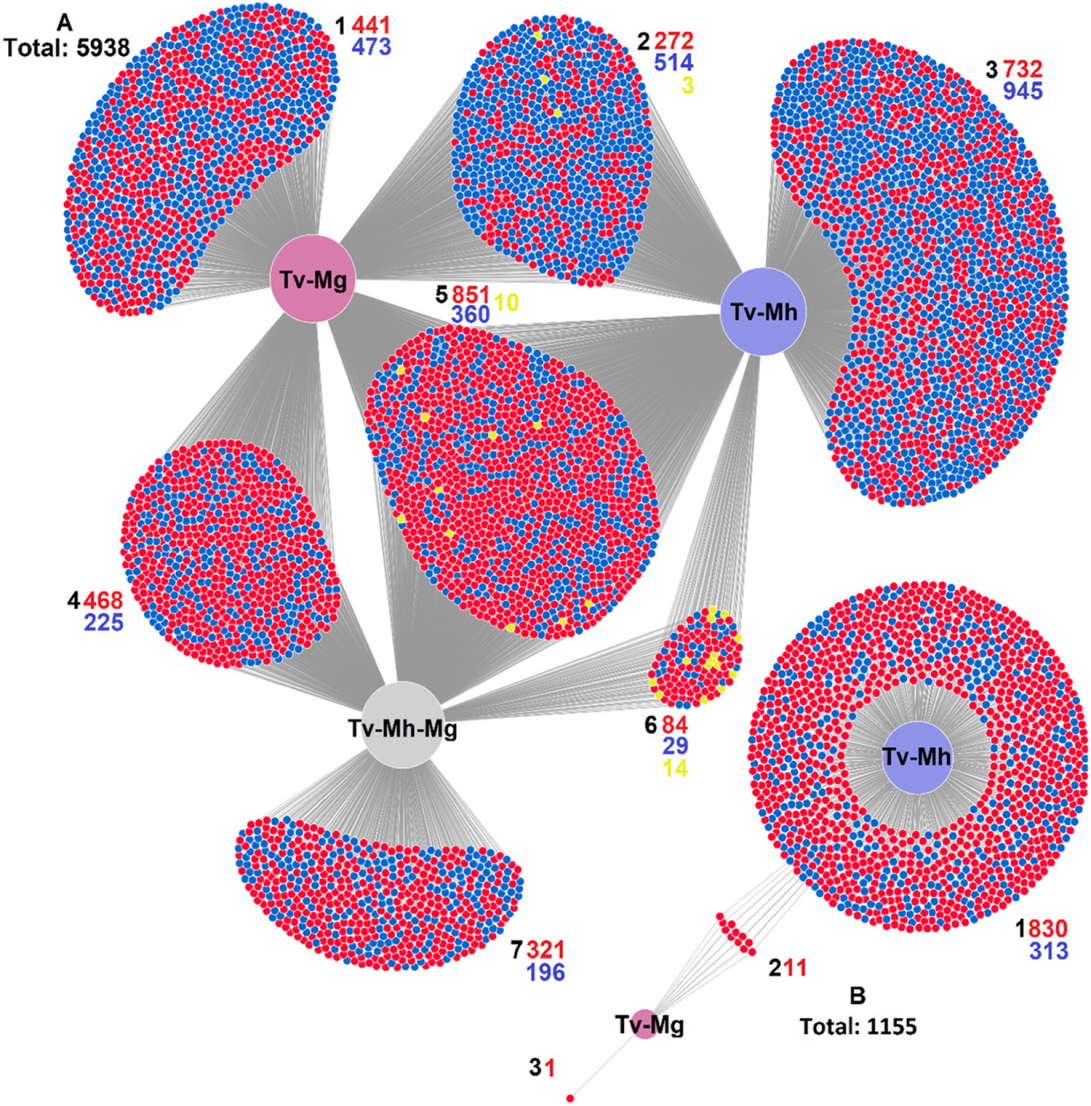
DiVenn diagrams depicting the extent of overlap of differentially expressed genes in pairwise comparisons between the different T. vaginalis-*Mycoplasma* symbioses. Large nodes symbolize specific T. vaginalis-*Mycoplasma* symbioses, and small nodes indicate individual genes. Edges link genes with comparisons under which they are significantly differentially expressed (FDR < 0.05) compared to the control TvSS-62iso. Small nodes are shown in red and blue to indicate upregulated and downregulated genes, respectively. Yellow nodes indicate disparity in the direction of regulation between multiple comparisons. Comparison nodes are in light blue for TvSS-62iso+Mh (Tv-Mh), mauve for TvSS-62Mg (Tv-Mg), and gray for TvSS-62Mg+Mh (Tv-Mh-Mg). Specific clouds/groupings of genes are labeled 1 to 6 in panel A and 1 to 3 in panel B. Separate DiVenn diagrams are shown for comparisons between either the control TvSS-62iso (Tv) and the different symbioses (A) or the TvSS-62Mg+Mh condition versus the two single symbioses using single-*Mycoplasma* conditions as the control (B). Numbers are used to label regions of gene nodes in black, and total gene counts for each region are shown in colors corresponding to the gene nodes. Total values indicate all genes in each DiVenn diagram.

The set of genes regulated in T. vaginalis in response to the presence of *Mycoplasma* largely overlapped between the TvSS-62iso+Mh, TvSS-62Mg, and TvSS-62Mg+Mh conditions compared with the control TvSS-62iso, corresponding to a core transcriptional response to the presence of either *Mycoplasma* species (Fig. 9A). Interestingly, this core set of genes appeared to be biased toward upregulation (Fig. 9). There were also large sets of genes that were uniquely regulated in response to individual conditions, indicating distinct transcriptional responses to *M. hominis*, “*Ca.* M. girerdii,” and the simultaneous presence of both mycoplasmas.

In order to examine the transcriptional response that was specific to the synergy of both mycoplasmas, differential expression was tested in the TvSS-62Mg+Mh condition versus the respective single-*Mycoplasma* conditions (Fig. 9B). Very few genes were differentially regulated in TvSS-62Mg+Mh compared with TvSS-62Mg alone, whereas there was a major transcriptional response resulting from the introduction of “*Ca*. M. girerdii” to the TvSS-62iso+Mh condition, consistent with a dominant effect of the presence of “*Ca*. M. girerdii.” Only 11 genes overlapped between these comparisons, which represent a gene set specific to the synergistic effect of both mycoplasmas compared with only a single species.

### Functional prediction of T. vaginalis differential gene expression.

**FIG 10:**
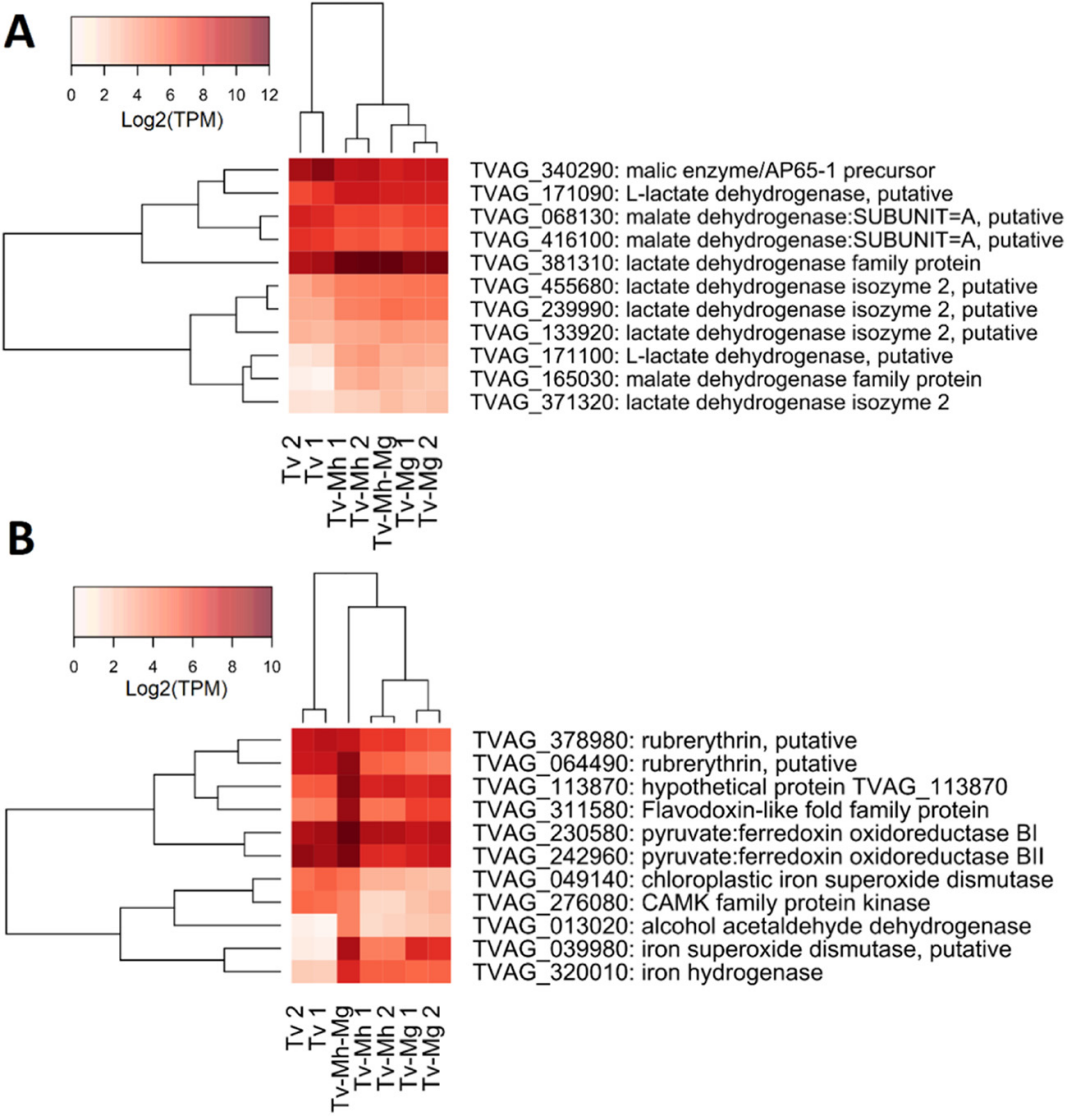
Heatmap of all genes significantly differentially expressed under both the TvSS-62iso+Mh and TvSS-62Mg conditions versus the TvSS-62Mg+Mh condition. Heatmaps show all significantly differentially expressed genes annotated with TCA cycle or malate metabolic process GO terms (A) and all genes significantly differentially expressed under both the TvSS-62iso+Mh (Tv-Mh) and TvSS-62Mg (Tv-Mg) conditions versus the TvSS-62Mg+Mh (Tv-Mh-Mg) condition (B). Expression is shown in units of log_2_ transcripts per million (TPM). CAMK, Ca2+/calmodulin-dependent protein kinase

Gene ontology (GO) enrichment analysis was used to summarize the functions of genes that were differentially expressed in response to the presence of *Mycoplasma* (Table 3). The core set of genes homodirectionally regulated in response to all three *Mycoplasma* conditions (Fig. 9A, region 5) was associated with a variety of metabolic responses. The catabolism of various amino acids, including threonine, aspartate, and glutamine family amino acids, increased in the presence of *Mycoplasma*. There was also an apparent increase in central energy metabolism via the hydrogenosome, indicated by processes such as “electron transport chain” and the misidentified “tricarboxylic acid cycle” (TCA cycle) (e.g., TVAG_165030 [malate dehydrogenase family protein]). We examined the expression of enzymes annotated as part of the TCA cycle, and the related malate metabolic process, in detail (Fig. 10A). Consistent with the enrichment analysis results, the majority (8 out of 11) of the genes were significantly upregulated in the presence of *Mycoplasma*. All the upregulated genes could be aligned well at the protein level with one another and T. vaginalis malate dehydrogenase (GenBank accession number AAC46986.1 [strain NIH-C1]) (99% identical to TVAG_253650 [strain G3]) investigated by previously by Wu and colleagues (49). All upregulated genes possessed the Arg91Leu mutation, which confers specificity to lactate. Among all TCA/malate metabolic process enzymes, this group also included the most highly expressed gene (TVAG_381310 [mean transcripts per million {TPM}, 2,600]) and the most highly upregulated gene under all *Mycoplasma* conditions versus the control (TVAG_165030 [log_2_ fold changes of 3.4, 2.6, and 3.0 under the TvSS-62iso+Mh, TvSS-62Mg, and TvSS-62Mg+Mh conditions, respectively]). The three downregulated genes aligned well with the Escherichia coli decarboxylating malic enzyme (RefSeq accession number NP_415996.2) (50), and two were predicted to be localized to the hydrogenosome by Burstein and colleagues (51). The single gene not predicted to be localized in hydrogenosomes (TVAG_068130) was truncated by approximately 296 amino acid residues at the N terminus compared with the other two and the E. coli enzyme, suggesting that it is a gene fragment. Overall, these results suggest that the presence of *Mycoplasma* may induce an increase in cytosolic lactate fermentation and a decrease in hydrogenosomal metabolism proceeding via malate catabolism. Finally, we also observed that there was an increase in “response to oxidative stress” with the two related entries “cellular oxidant detoxification” and “response to oxidative stress” (Table 3).

**TABLE 3:** The most significantly enriched GO biological process terms among genes regulated in response to all *Mycoplasma* symbioses^*a*^

Description	No. of expressed genes^*b*^	No. of DE genes^*c*^	*P* value	Direction of regulation
Glycerolipid biosynthetic process	30	9	2.28E−06	Negative
Lipid biosynthetic process	72	12	2.52E−06	
Phospholipid biosynthetic process	45	9	2.70E−05	
Phosphatidylcholine biosynthetic process	4	4	0.00051	
Microtubule-based movement	149	12	0.000651	
Cellular lipid catabolic process	15	5	0.00117	
*S*-Adenosylmethionine biosynthetic process	6	3	0.0212	
Glycerol biosynthetic process from pyruvate	8	3	0.026	
Short-chain fatty acid catabolic process	8	3	0.0266	
Response to lipid	8	3	0.0272	
Steroid metabolic process	8	3	0.0291	
Alditol biosynthetic process	8	3	0.0314	
Triglyceride biosynthetic process	8	3	0.0322	
Fatty acid catabolic process	10	3	0.0346	
Cellular response to glucose stimulus	8	3	0.036	
Propionate catabolic process	8	3	0.0371	
Cellular glucose homeostasis	8	3	0.0382	
Iron-sulfur cluster assembly	27	12	5.53E−07	Positive
Protein phosphorylation	1,056	74	3.00E−06	
Cofactor metabolic process	97	16	6.61E−05	
Cellular oxidant detoxification	26	9	9.20E−05	
Cellular response to toxic substance	26	9	9.42E−05	
Aerobic respiration	28	9	0.000143	
Oxaloacetate metabolic process	21	8	0.000179	
Glutamine family amino acid metabolic process	24	8	0.000352	
Drug metabolic process	77	13	0.000382	
Tricarboxylic acid cycle	26	8	0.000543	
Malate metabolic process	26	8	0.000553	
Protein dephosphorylation	301	27	0.000638	
NADH metabolic process	30	8	0.00122	
Aspartate family amino acid catabolic process	9	5	0.00252	
Response to oxidative stress	35	8	0.00289	
Negative regulation of transcription, DNA templated	61	10	0.00417	
Electron transport chain	50	9	0.00476	
Negative regulation of transcription from RNA polymerase II promoter	30	7	0.00641	
Negative regulation of RNA biosynthetic process	70	10	0.00986	
Threonine catabolic process	8	4	0.0146	

a The top 20 lowest *P* value terms are shown for upregulated and downregulated genes, ranked by increasing *P* values.

b Total number of genes associated with the GO term with detected expression in this experiment.

c Number of differentially expressed (DE) genes associated with the GO term.

The results from KEGG enrichment analysis were largely in agreement with those from the GO term enrichment analysis and also highlighted upregulated enzymes potentially involved in the synthesis (such as TVAG_388260 [UDP-glucose pyrophosphorylase]) and utilization (such as TVAG_185930 [alpha amylase] and TVAG_222040 [4-alpha-glucanotransferase]) of cellular glycogen stores, under the category “starch and sucrose metabolism” (Table S4). Intriguingly, processes downregulated in response to *Mycoplasma* included pathways involved in the biosynthesis and catabolism of lipids and motility (Table 3). To investigate any significance of changes in lipid metabolism among the three species, we examined differences in KEGG-annotated lipid metabolic enzymes. “*Ca*. M. girerdii” possesses several enzymatic functions involved in glycerophospholipid biosynthesis apparently absent in T. vaginalis, including acyl phosphate:glycerol-3-phosphate acyltransferase, cardiolipin synthase, and phosphate acyltransferase. *M. hominis* possesses only one annotated lipid metabolism-associated enzyme absent in T. vaginalis, phosphate acyltransferase.

10.1128/mbio.00918-22.7 TABLE S4 Core Myco KEGG annotations. Shown are upregulated enzymes potentially involved in the synthesis and utilization of cellular glycogen stores, under the category “starch and sucrose metabolism.” Download Table S4, XLSX file, 0.01 MB. Copyright © 2022 Margarita et al. 2022 Margarita et al. https://creativecommons.org/licenses/by/4.0/ This content is distributed under the terms of the Creative Commons Attribution 4.0 International license.

The enrichment analysis results also indicated that the regulatory responses to *Mycoplasma* involve transcription-level control and protein phosphorylation and dephosphorylation (Table 3).

Notably, the TvSS-62Mg and double-symbiosis conditions showed highly similar transcriptional profiles (Fig. 9B, region 2). In addition, the set of 11 genes specific to this condition versus the individual symbioses encoded reactive oxygen species (ROS) detoxification enzymes and hydrogenosomal energy generation enzymes (Fig. 10B).

### Impact of symbiosis on ADH pathway gene expression.

**FIG 11:**
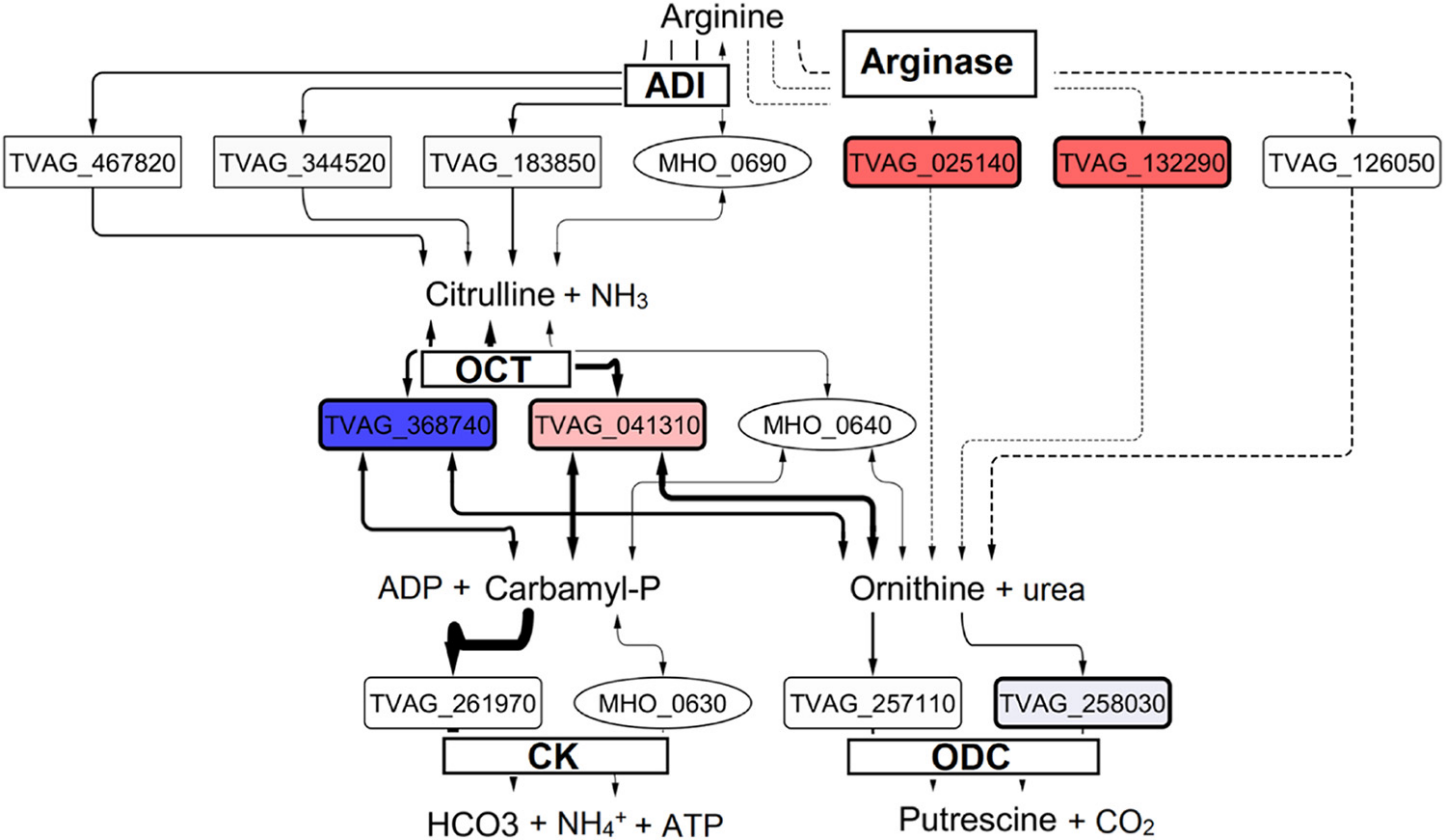
Expression of arginine dihydrolase (ADH) pathway genes. T. vaginalis hydrogenosomal and cytoplasmic enzymes and *M. hominis* enzymes are enclosed in squared rectangles, rounded rectangles, and ovals, respectively. T. vaginalis enzymes that are significantly differentially expressed under the Tv-Mh versus Tv conditions are bordered in bold, and the fill color is scaled from blue (downregulated) to red (upregulated) according to their log_2_ fold changes in this comparison. For T. vaginalis enzymes, the arrow thickness is scaled to their expression level in TPM. Dashed lines for arginase indicate uncertainty as to whether activity for this enzyme is present in T. vaginalis (68, 69). ADI (Arginine deiminase); CK (Carbamate kinase); OCT (Ornithine carbamyltransferase); ODC (ornithine decarboxylase)

We investigated the impact of *Mycoplasma* symbiosis on arginine dihydrolase (ADH) pathway enzyme expression due to its shared importance as a means of energy generation in T. vaginalis and *M. hominis* (22) (Fig. 11). The majority of T. vaginalis ADH enzymes were not significantly regulated. However, intriguingly, for ornithine carbamoyl transferase (OCT) (the most highly expressed enzyme of the pathway), the two homologs showed opposite mild but significant regulatory profiles in response to *Mycoplasma*. TVAG_041310 was upregulated with log_2_ fold changes of 0.8, 1.0, and 1.1 under the TvSS-62iso+Mh (Tv-Mh), TvSS-62Mg (Tv-Mg), and TvSS-62Mg+Mh (Tv-Mh-Mg) conditions versus the control, whereas TVAG_368740 was downregulated with corresponding log_2_ fold change values of −1.3, −1.4, and −0.9, respectively. Based on the reconstructed open reading frame (ORF) from the RNA-Seq data, the two enzymes were 97% identical at the protein level, with 8 mismatching residues. However, none of the mutations coincided with highly conserved or putative functionally important residues in published data on OCT enzymes (52). The two enzymes were 92% identical at the nucleotide level, with mutations present within all 50-bp windows, and thus were likely easily distinguished during the alignment of the 150-bp RNA-Seq reads. Interestingly, the sum of the TPM values for the two enzymes dropped slightly from a mean of 219 to 181 under the Tv-Mh condition versus the control, possibly corresponding to a decrease in enzyme activity. In contrast, the corresponding values were 201 and 217 under the Tv-Mg and Tv-Mh-Mg conditions, respectively. Two putatively annotated arginase enzymes, which are not considered part of the ADH pathway, were also significantly upregulated by a similar magnitude under all *Mycoplasma* conditions. The strongest regulation was for the putative arginase TVAG_025140, with log_2_ fold changes of 2.8, 3.6, and 4.4 under the Tv-Mh, Tv-Mg, and Tv-Mh-Mg conditions versus the control, respectively.

### Analysis of genes potentially implicated in T. vaginalis pathobiology.

**FIG 12:**
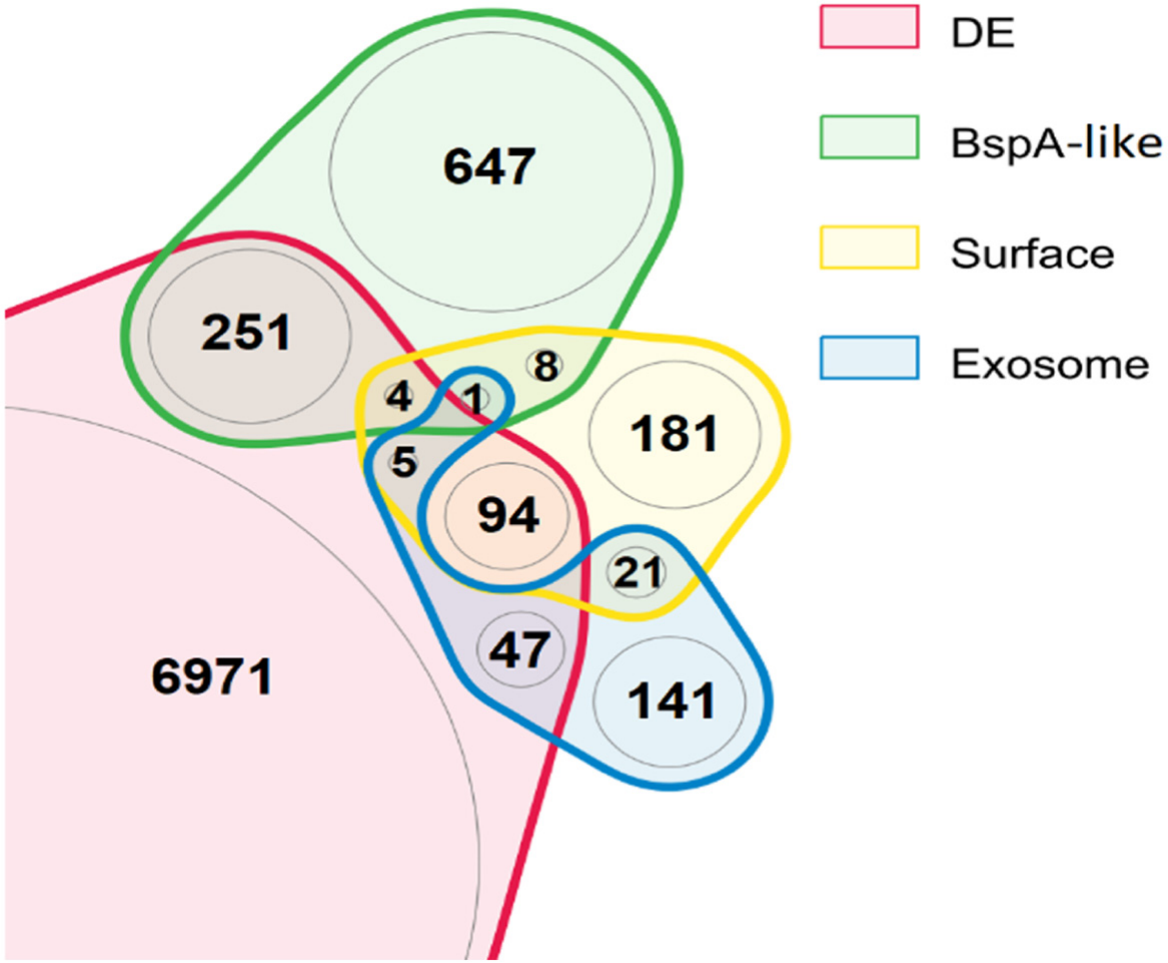
Differential expression of pathobiology-associated genes during T. vaginalis-*Mycoplasma* coculture. The Venn diagram shows the overlap in gene sets differentially expressed (DE) under any *Mycoplasma* condition, annotated as BspA (37), experimentally verified surface proteins (54), and experimentally verified exosome proteins (55). Only the region showing the total list of differentially expressed genes is cropped.

**FIG 13:**
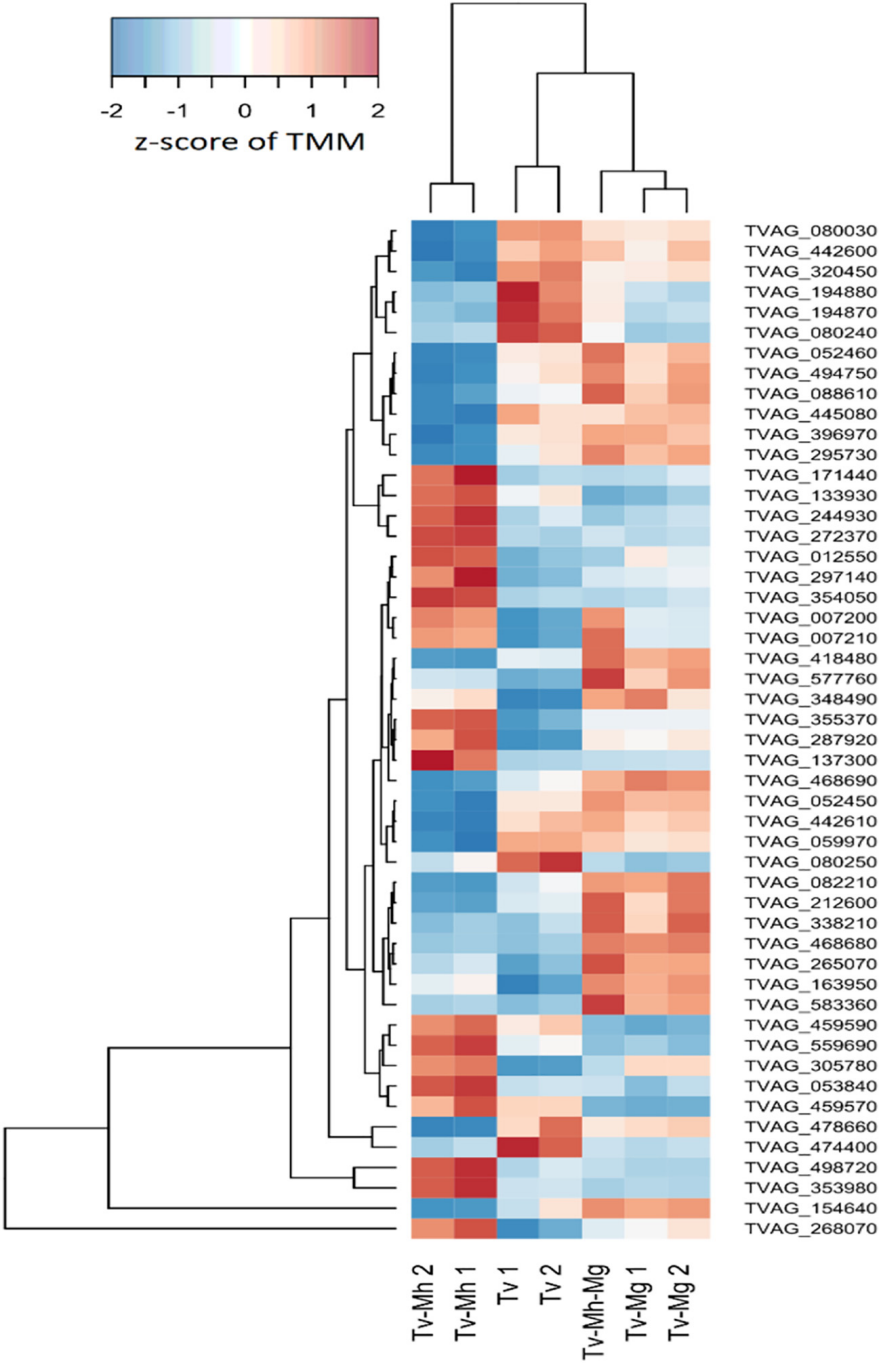
Differential expression of T. vaginalis annotated BspA genes during coculture with *Mycoplasma*. A heatmap of annotated T. vaginalis BspAs (37) with the top 50 lowest *P* values for differential expression under any *Mycoplasma* condition is shown. Expression units are z-scaled TMM.

**FIG 14:**
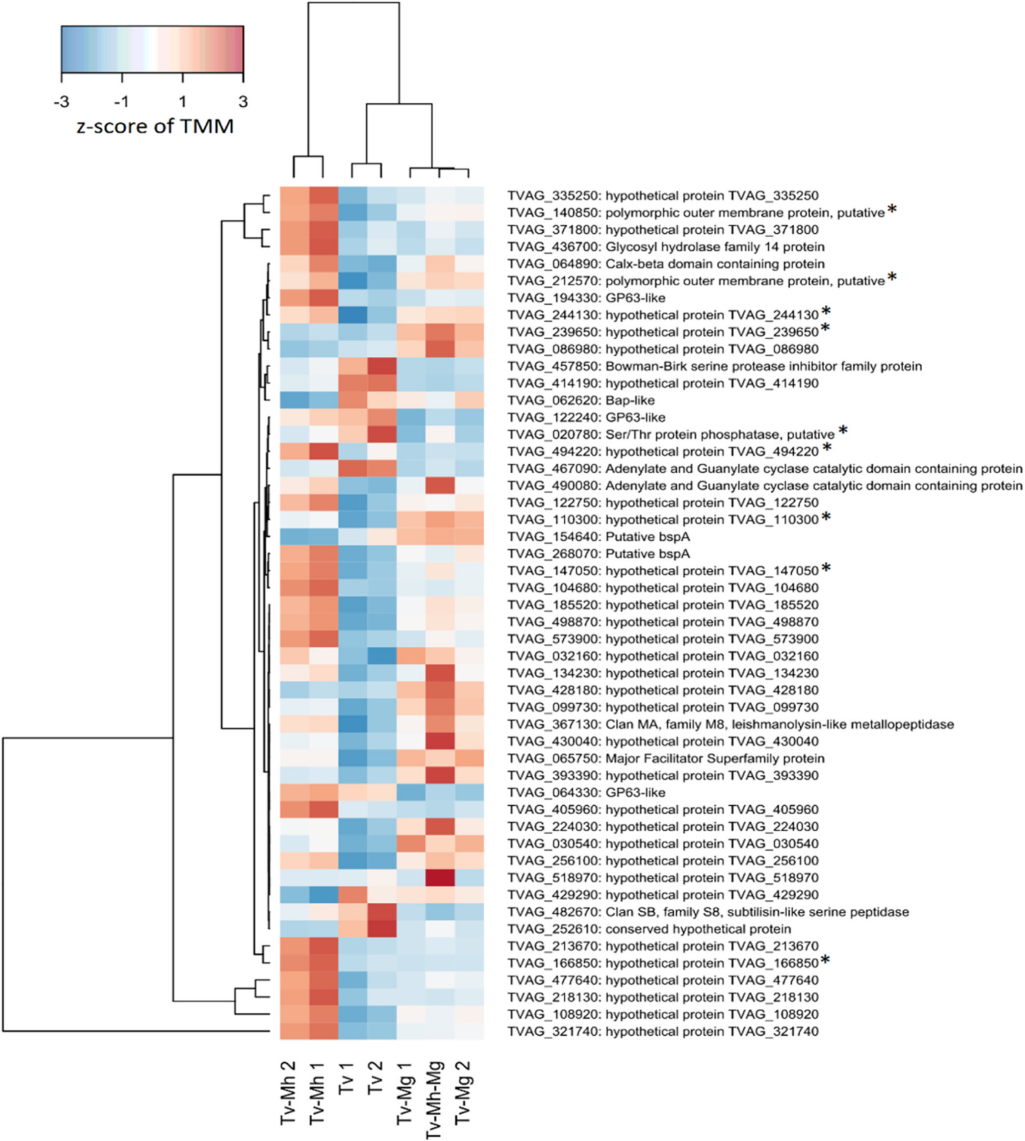
Differential expression of T. vaginalis surface protein-coding genes during coculture with *Mycoplasma*. A heatmap of experimentally verified surface protein-coding genes (54) with the top 50 lowest *P* values for differential expression under any *Mycoplasma* condition is shown. Expression units are z-scaled TMM. Genes with significantly increased abundance among more adherent T. vaginalis strains (54) are marked with an asterisk.

To investigate the regulation of genes potentially implicated in T. vaginalis pathobiology, we examined the expression of annotated surface proteins, including TvBspA-like proteins that can mediate cell-cell interactions (37, 53), and experimentally verified exosomal proteins, implicated in adhesion to host cells and immunomodulation (54–56) (Fig. 12). Out of a total of 911 TvBspA-like proteins, 255 were significantly differentially regulated under at least one of the tested *Mycoplasma* symbioses, and similarly, 103 out of 314 experimentally verified surface proteins (EVSPs) and 52 out of 215 exosome proteins were also significantly modulated. Notably, TvBspA-like genes, surface proteins, and exosome proteins were significantly modulated as gene sets under each *Mycoplasma* condition versus the control (false discovery rate [FDR] < 0.001). However, gene set modulation in a particular direction, either upregulation or downregulation, was not significant (FDR > 0.05), indicating no clear global directional response (Table 4). Many EVSPs, including some TvBspA-like proteins, showed expression profiles that were specific to each *Mycoplasma* species (Fig. 13 and 14). An overall positive regulation of exosome proteins under the TvSS-62Mg+Mh condition versus the control was near the threshold for significance (*P* value of 0.075).

**TABLE 4:** Adjusted *P* values for gene set testing of genes implicated in T. vaginalis pathobiology^*a*^

Gene set (reference)	Adjusted *P* value					
	Nondirectional DE			Upregulated		
	Tv-Mh	Tv-Mg	Tv-Mh-Mg	Tv-Mh	Tv-Mg	Tv-Mh-Mg
Surface proteins (54)	4.48 × 10^−4^	9.77 × 10^−7^	5.35 × 10^−4^	0.245	0.761	0.493
BspA-like proteins (37)	7.02 × 10^−6^	9.21 × 10^−6^	5.15 × 10^−5^	0.612	0.761	0.526
Exosome proteins (55)	3.49 × 10^−5^	6.21 × 10^−4^	5.36 × 10^−4^	0.245	0.111	0.0752

a Values are given for each of the *Mycoplasma* conditions versus the control T. vaginalis SS-62iso, which is *Mycoplasma* free. Tv-Mh, TvSS-62iso+Mh; Tv-Mg, TvSS-62Mg; Tv-Mh-Mg, TvSS-62Mg+Mh.

**FIG 15:**
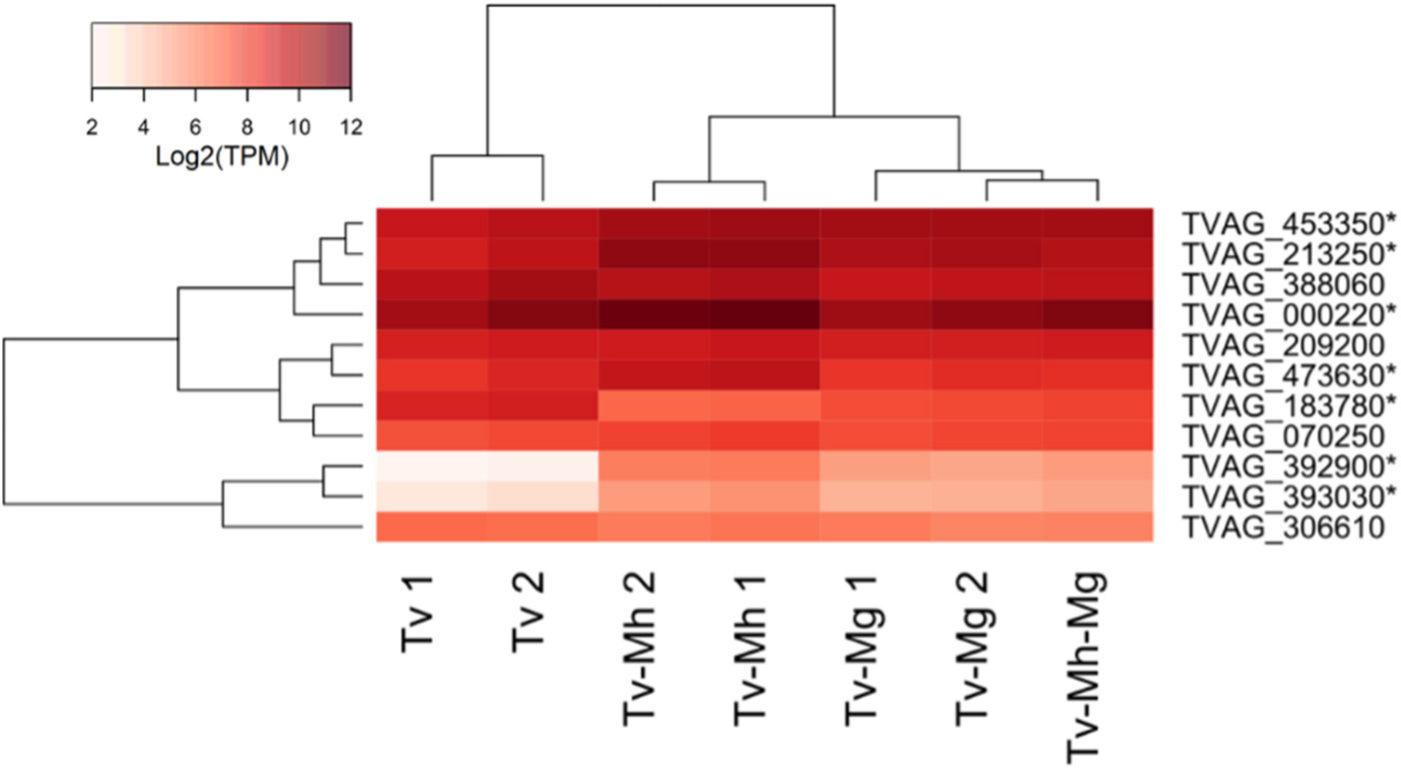
Heatmap of genes encoding saposin-like (TvSAPLIP) proteins significantly differentially expressed under TvSS-62iso+Mh, TvSS-62Mg, and TvSS-62Mg+Mh conditions versus the TvSS-62iso condition. The heatmap shows 7 out of the 11 transcripts encoding candidate TvSAPLIP proteins significantly differentially expressed under TvSS-62iso+Mh (Tv-Mh), TvSS-62Mg (Tv-Mg), and TvSS-62Mg+Mh (Tv-Mh-Mg) conditions versus the TvSS-62iso condition. Expression is shown in units of log_2_ transcripts per million (TPM).

Of particular interest, 9 out of 11 genes significantly upregulated among more adherent T. vaginalis strains (54) were significantly regulated in response to *Mycoplasma*, 8 of which were upregulated under one or more of the *Mycoplasma* conditions compared with the control (Fig. 13 and 14; Table S5). These included two members of the polymorphic outer membrane (Pmp) protein family (57). One Pmp entry (TVAG_140850) was shown experimentally to boost the binding of parasites to vaginal epithelial cells when overexpressed (53). These are members of a larger family of hypothetical proteins in T. vaginalis (155 members [see reference 57]) related to the bacterial Bap (for biofilm-associated protein) surface proteins from various bacterial species and the InlB protein, a member of the internalin protein family in *Listeria*, which can mediate bacterial biofilm formation and binding to and internalization in eukaryotic host cells (58, 59). At least five additional members of this Bap-like protein family were also significantly upregulated in the presence of either of the *Mycoplasma* species (TVAG_359980, TVAG_238790, TVAG_238780, TVAG_238800, and TVAG_200680) (Table S3). We also investigated the modulation of the transcripts encoding the saposin-like protein family members (SAPLIPs), which potentially mediate the pore-forming activity underlying hemolysis (46, 57). Of the 11 transcribed SAPLIP genes, 7 transcripts were significantly upregulated (Fig. 15).

10.1128/mbio.00918-22.8 TABLE S5 Upregulated proteins and genes. Genes significantly upregulated among more adherent T. vaginalis strains in response to *Mycoplasma* are shown. Download Table S5, XLSX file, 0.01 MB. Copyright © 2022 Margarita et al. 2022 Margarita et al. https://creativecommons.org/licenses/by/4.0/ This content is distributed under the terms of the Creative Commons Attribution 4.0 International license.

### Expression of “*Ca.* M. girerdii” and *M. hominis* genes.

Despite the low sequence coverage for *Mycoplasma* transcripts (Fig. 6), expression was detected for 495 out of 652 *M. hominis* genes (76%) and 553 out of 563 “*Ca*. M. girerdii” genes (98%) across all *Mycoplasma*-containing samples (Table S6). The ADH pathway was highly active in *M. hominis*, with the 3 contributing enzymes, arginine deiminase, ornithine carbamoyl transferase, and carbamate kinase, all included among the 25 ORFs with the highest TPM values (Table S5). In “*Ca*. M. girerdii,” various putative amino acid transporters, enzymes involved in amino acid catabolism (alanine dehydrogenase [B1217_0546], serine dehydratase [B1217_0101], and cysteine desulfidase [B1217_0039]), and the full annotated glycolytic pathway had high numbers of mapped reads, suggesting energy generation via these pathways. Pyruvate formate-lyase (B1217_0461) and its potentially associated autonomous glycyl radical cofactor (B1217_0541) were among the most abundant transcripts, suggesting that anaerobically adapted glycolysis was active. Concurrent with numerous T. vaginalis BspA-like-encoding genes being transcribed, including a number of entries modulated by the presence of “*Ca*. M. girerdii” (Fig. 12), we detected “*Ca*. M. girerdii” expression for 24 out of a total of 26 (27) annotated BspA-like proteins. The BspA-like gene B1217_0328 was also among the most highly expressed genes (15th most transcribed gene) (Table S5). Notably, the “*Ca*. M. girerdii” gene B1217_0162, a putative 2′,3′-cyclic nucleotide 2′-phosphodiesterase (catalyzing KEGG reaction K01119), was also highly expressed (45th most transcribed gene), in parallel with the T. vaginalis adenylate and guanylate cyclase-encoding genes TVAG_365230 and TVAG_451920, which were among the most highly upregulated genes in the presence of “*Ca*. M. girerdii” and putatively generate the cyclic nucleotide substrate for the *Mycoplasma* phosphodiesterase enzyme. Sequence analysis of B1217_0162 predicted that it is a candidate surface protein anchored to the extracellular face of the plasma membrane via an N-terminal lipoprotein signal peptide.

10.1128/mbio.00918-22.9 TABLE S6 *Mycoplasma* genes expressed. Expression data for “*Ca*. M. girerdii” and *M. hominis* genes are shown. Download Table S6, XLSX file, 0.1 MB. Copyright © 2022 Margarita et al. 2022 Margarita et al. https://creativecommons.org/licenses/by/4.0/ This content is distributed under the terms of the Creative Commons Attribution 4.0 International license.

### Variability of “*Ca.* M. girerdii” MOIs in TvSS-62Mg under stress conditions.

**FIG 16:**
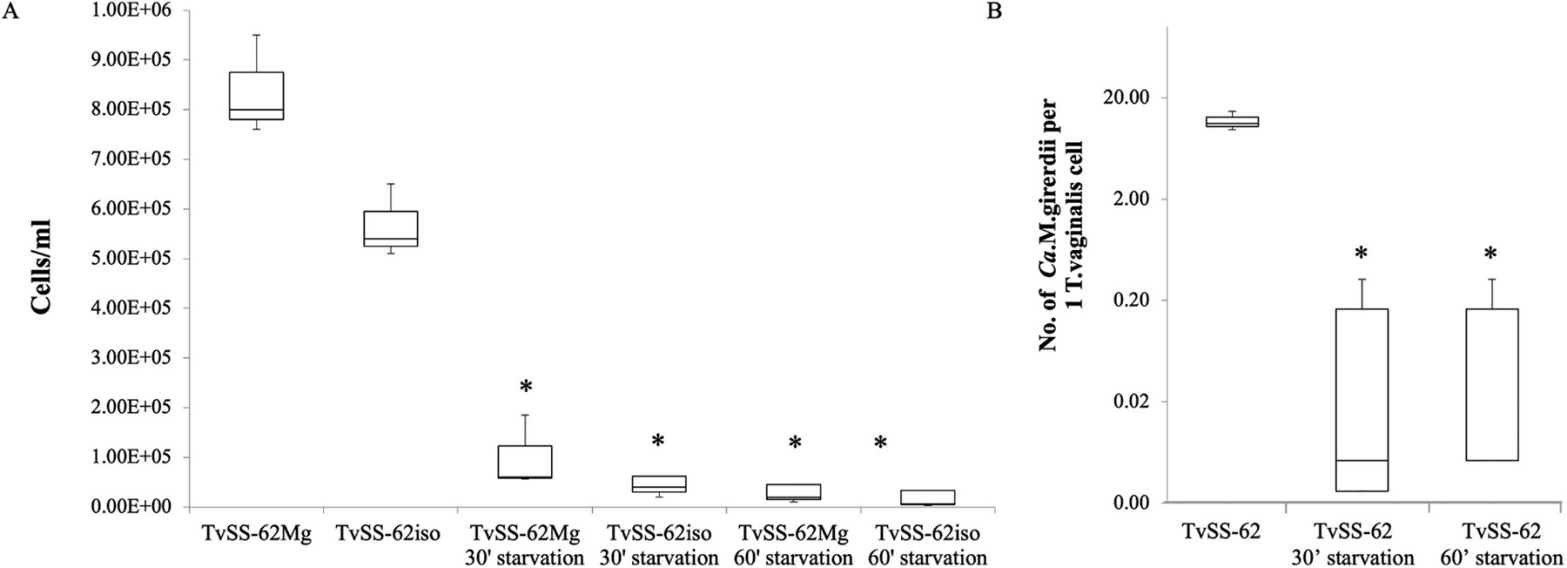
Impact of nutritional stress on T. vaginalis-“*Ca*. M. girerdii” symbiosis. (A) Cultures from isolate TvSS-62Mg (naturally infected with “*Ca*. M. girerdii”) and TvSS-62iso (experimentally cleaned from “*Ca*. M. girerdii”) were grown in complete medium for 24 h, used as controls for comparison with TvSS-62Mg and TvSS-62iso exposed to PBS for either 30 min or 60 min, and then grown for 24 h in complete medium, and counts of T. vaginalis cells were performed from triplicates. Data were analyzed by Student’s *t* test, and * indicates a *P* value of <0.05 for T. vaginalis cultures exposed to normal medium compared to cells exposed for 30 or 60 min to PBS. (B) The impact of the TvSS-62Mg symbiosis exposed to control or PBS medium on the MOI of “*Ca*. M. girerdii” in T. vaginalis was measured by qPCR on the set of samples shown in panel A. Either starvation condition massively decreased the mean MOI value of “*Ca*. M. girerdii” in trichomonad cells compared with the control, indicating a high sensibility of the bacteria to the tested environmental change. Bars represent the means ± SD, and the boxes represent the number of T. vaginalis cells in 1 mL of culture (A) and the number of “*Ca*. M. girerdii” bacteria per 1 trichomonad cell (B) from three independent growth experiments. Statistical significance was tested by Student’s *t* test, and * indicates significant (*P* < 0.05) variations in terms of the mean MOI value of “*Ca*. M. girerdii” per T. vaginalis cell.

We observed that the presence of “*Ca.* M. girerdii” in symbiosis with T. vaginalis (strain TvSS-62Mg) did not influence the viability of the parasite during the extreme nutrient stress for the tested incubation times (phosphate-buffered saline [PBS] for 30 min and 60 min) (Fig. 16A). Notably, these starvation conditions strongly influence the “*Ca.* M. girerdii” mean MOI bacterium/trichomonad ratio, dramatically decreasing from 12:1 in standard Diamond’s TYM medium down to 1:9 and 1:750 following 30-min and 60-min starvation conditions, respectively (Fig. 16B). These results indicate an increase of xenophagy of the bacteria by T. vaginalis in response to the tested stress conditions, a direct impact of the stress conditions on the bacteria, or a combination of these two options. A similar phenomenon was observed from limiting dilution experiments with TvSS-62Mg where the presence of “*Ca.* M. girerdii” was detected until a dilution of 12.5 T. vaginalis cells/well in 100 μL of medium.

### *Mycoplasma* in symbiosis with T. vaginalis increases both parasite hemolytic properties and adherence to human epithelial cells.

**FIG 17:**
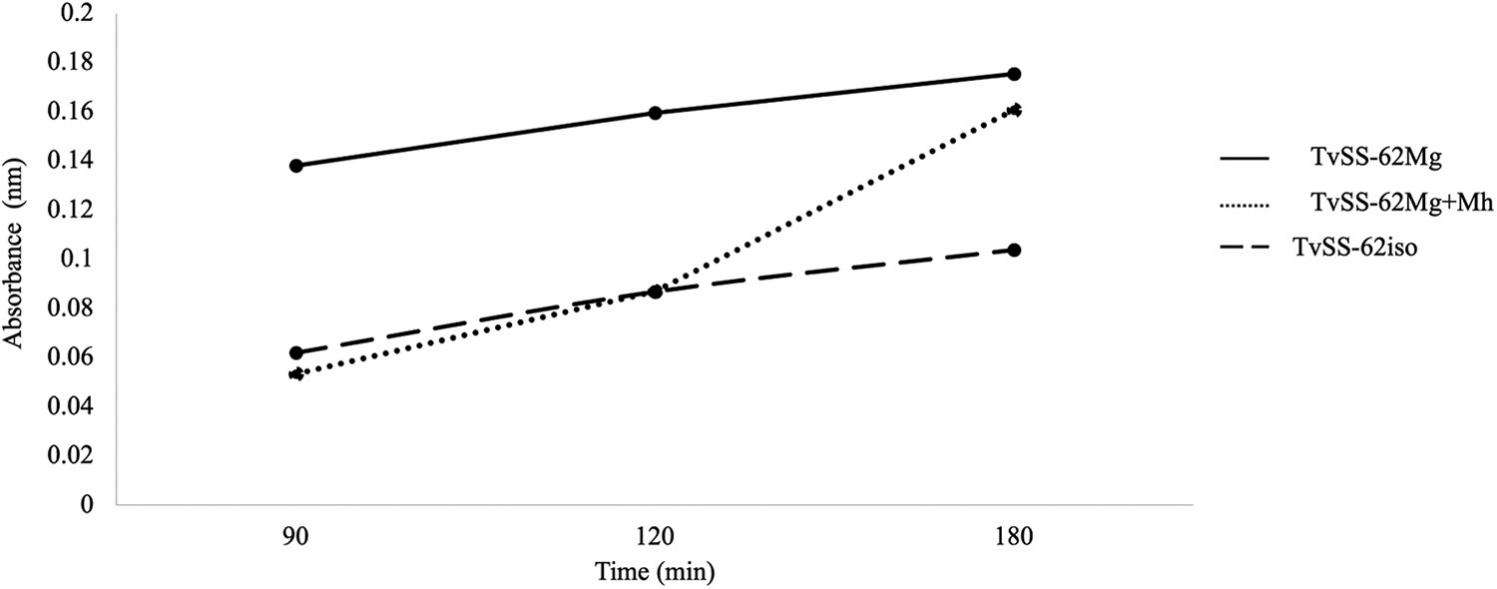
Influence of *Mycoplasma* species on the hemolytic activity of T. vaginalis. The hemolytic activity exerted by “*Ca*. M. girerdii” alone and associated with *M. hominis* in T. vaginalis SS-62 was compared with that of T. vaginalis SS-62iso (experimentally cleaned from “*Ca*. M. girerdii”), evaluating hemoglobin released by human RBCs through spectrophotometric analysis (reading at a 546-nm absorbance). The values are expressed as hemoglobin released by RBCs upon contact with pathogens and represent averages and standard deviations (error bars) from three independent experiments. Statistical significance was investigated by Student’s *t* test, and * indicates significant (*P* < 0.05) variations compared to parasites without *Mycoplasma* species.

The observed significant influence of “*Ca.* M. girerdii” and *M. hominis* on the transcriptome of the protist led us to evaluate with two different cell-based assays the potential modulation of the two *Mycoplasma* species on the pathobiology of the parasite. Using a hemolysis assay (60), we examined the amount of hemoglobin released by human erythrocytes (RBCs) upon contact with TvSS-62iso (mycoplasma-free T. vaginalis), TvSS-62Mg (T. vaginalis naturally infected by “*Ca.* M. girerdii”), and TvSS-62Mg+Mh (T. vaginalis experimentally infected by *M. hominis*) over three time points, 90, 120, and 180 min. Similar to the data for T. vaginalis strain G3 associated with *M. hominis* (22), the protists infected by “*Ca.* M. girerdii” and with double infection were both characterized by higher hemolytic activities than for the mycoplasma-free isogenic T. vaginalis strain at the 180-min time point (*P* < 0.05) (Fig. 17).

**FIG 18:**
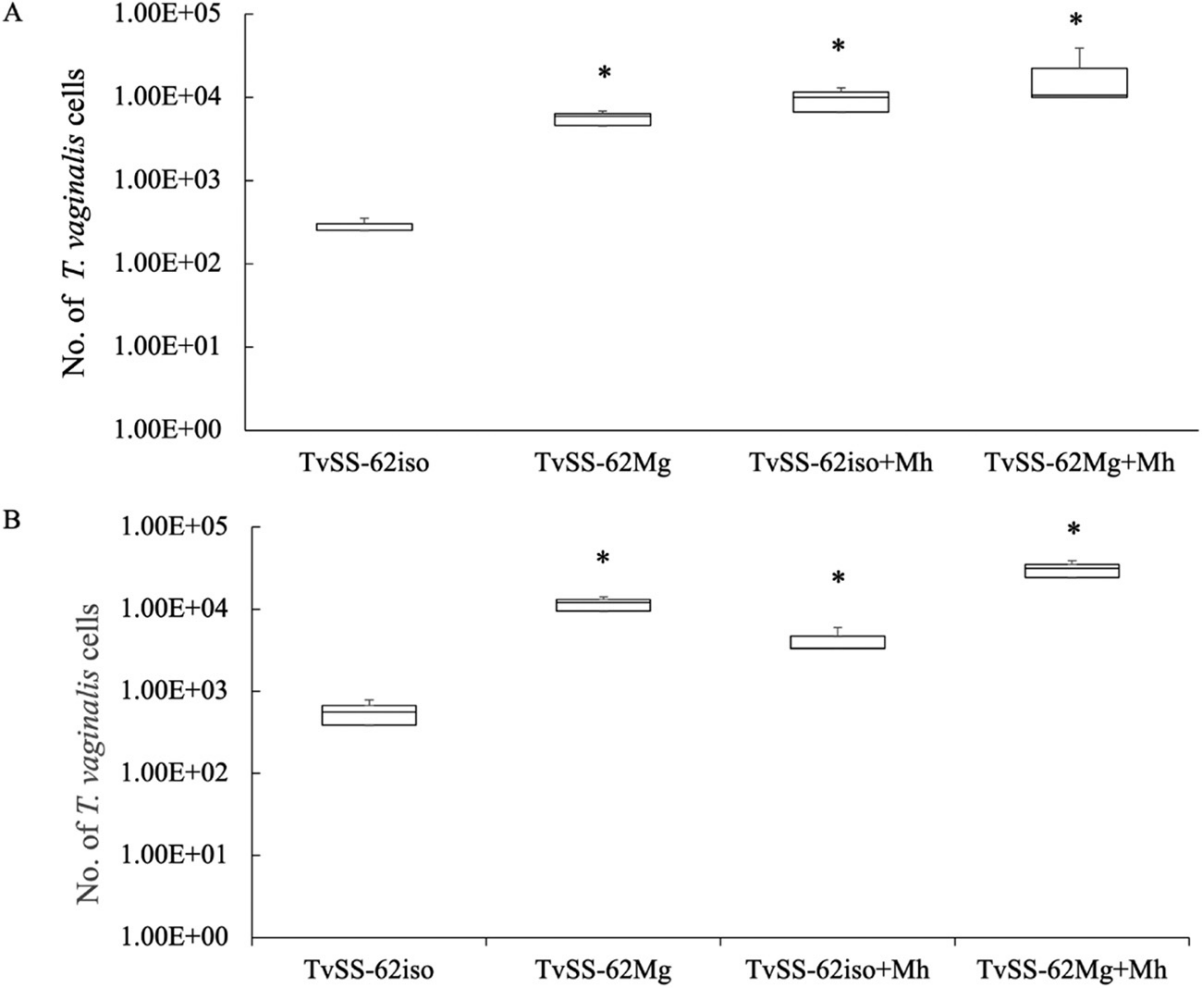
*Mycoplasma* increases the adherence of T. vaginalis to epithelial cells. Adherence to NOK cells (A) and HeLa cells (B) of T. vaginalis isogenic strains (TvSS-62iso, experimentally mycoplasma cleaned; TvSS-62Mg, naturally “*Ca*. M. girerdii” infected; and TvSS-62iso+Mh and TvSS-62Mg+Mh, experimentally infected by *M. hominis*) was evaluated, and the number of trichomonad cells attached to epithelial cells after a 30-min incubation was determined by qPCR. Data obtained from two experiments performed in triplicate show that the presence of “*Ca*. M. girerdii” and *M. hominis* in T. vaginalis statistically influences adhesion, increasing the number of cells attached to the monolayer (*, *P* < 0.05).

In agreement with the *Mycoplasma*-associated significantly increased amounts of transcripts for a number of genes encoding surface proteins mediating binding to the host cell (48), parasite binding to human NOK and HeLa cells after a 30-min incubation was significantly upregulated (~10-fold) in the presence of either *Mycoplasma* species, or the combination, compared to the isogenic strain TvSS-62iso (Fig. 18).

These results support the hypothesis suggested by the RNA-Seq data that the presence of *M. hominis* and “*Ca.* M. girerdii” influences positively both the capacities for T. vaginalis hemolysis and adhesion to host epithelial cells, two important features of T. vaginalis pathobiology (33, 54, 60).

## DISCUSSION

The ability of T. vaginalis to act in concert with endosymbiotic bacteria and viruses in the vaginal environment is an intriguing aspect of protozoan pathobiology (13, 21, 33, 61) and represents a fascinating and unique case of comorbidity from distinct microbes involved in different combinations of endosymbiosis, which can involve various combinations of up to two *Mycoplasma* species and up to four TVVs. Notably, multimorbidities are increasingly recognized to represent significant contributors to both mortality and morbidity rates during pregnancy (34), and the acquisition of T. vaginalis during gestation represents an additional risk for adverse pregnancy outcomes (61, 62). The parasite-*Mycoplasma*-TVV consortia could activate an excessive inflammatory response upon the release of TVV virions and *Mycoplasma* cells (21) after treatment with metronidazole, potentially complicating the outcome of pregnancy (15, 20, 21, 63). Furthermore, metagenomic investigations and 16S rRNA microbial surveys have shown that several vaginal bacterial communities are associated with trichomoniasis (26, 27, 64). The vaginal microbiota of women with trichomoniasis is characterized by an abundance of *M. hominis* (20, 61) and by the presence of an uncultured bacterium named “*Ca.* M. girerdii” (26, 27). In the current study, we developed an *in vitro* model of coinfection demonstrating the endosymbiotic nature of the relationship between the two *Mycoplasma* species and T. vaginalis.

Epidemiological data on “*Ca.* M. girerdii” analyzed in this work identified a prevalence of 61% among clinical T. vaginalis isolates derived from vaginal swabs from 75 Italian patients affected by acute trichomoniasis. This was consistent with the previously published prevalence of 44% (63 patients) to 63% (30 patients) for “*Ca.* M. girerdii” in vaginal samples of women clinically diagnosed with trichomoniasis in the United States (26, 27). A BLASTN search of the NCBI database with the 16S rRNA gene from “*Ca.* M. girerdii” (strain VCU_M1) identified hits with 100% identity from sequences resulting from various vaginally derived samples from China (e.g., GenBank accession numbers LC272065.1 and LC554418.1), and more recently, cultures of T. vaginalis associated with either of the mycoplasma species investigated were also characterized from T. vaginalis clinical isolates from Chinese patients (39). These different data suggest that the presence of “*Ca.* M. girerdii” is likely to be observed worldwide and is likely relevant for T. vaginalis-infected patients across most, including Caucasian, African-American, and Han people, if not all, ethnic groups across the globe.

Notably, our study shows that T. vaginalis is rarely uniquely associated with “*Ca*. M. girerdii” as 56% of T. vaginalis clinical isolates are naturally associated with both “*Ca.* M. girerdii” and *M. hominis*, with only 5% of T. vaginalis isolates analyzed in this work being associated with “*Ca*. M. girerdii” alone. Moreover, only 11% of T. vaginalis isolates from the investigated cohort of patients from Italy were mycoplasma free. Consistent with these data, a broad 16S rRNA gene survey on vaginal swabs from mainly African-American women (27) also identified both mycoplasma species from the same patient, with the majority (67%) of T. vaginalis infections associated with one or both mycoplasma species, indicating that the T. vaginalis association with “*Ca*. M. girerdii” and/or *M. hominis* is globally distributed across communities. These data strongly support the hypothesis that T. vaginalis is able to establish a stable relationship with “*Ca*. M. girerdii” alone and in association with *M. hominis*. Furthermore, this symbiosis might be more robust *in vivo* since based on the 16S rRNA survey data, “*Ca*. M. girerdii” was shown to be able to dominate the bacterial portion of the vaginal microbiome in the presence of *M. hominis* and T. vaginalis (27) (Fig. 1).

These results led us to investigate the MOIs of “*Ca.* M. girerdii” among different T. vaginalis isolates and to evaluate whether the presence of one mycoplasma species might influence the other under controlled *in vitro* growth conditions. Our data based on quantitative real-time PCR demonstrated that the ratio of “*Ca*. M. girerdii” to trichomonad cells in four isolates ranged from 4:1 to 18:1 (mean, 11:1) when “*Ca*. M. girerdii” is associated exclusively with T. vaginalis. Notably, in all T. vaginalis isolates in symbiosis grown *in vitro* with both mycoplasmas, the estimated MOI of “*Ca*. M. girerdii” per trichomonad cell was significantly lower (mean value of 1:19 bacteria per T. vaginalis cell). This was in contrast to the proportion of reads that mapped to 16S rRNA genes in vaginal swabs, with the majority of samples characterized by a majority of reads attributed to “*Ca*. M. girerdii” compared to *M. hominis* in T. vaginalis-infected patients (Fig. 1; see also Table S1 in the supplemental material) (27). While there are biases in proportional data from 16S rRNA surveys (41, 65), this suggests at face value that the biomass of “*Ca*. M. girerdii” is typically higher than that of *M. hominis* when both bacteria are present in T. vaginalis-infected women (Fig. 1B and D). Considering that both mycoplasmas are predicted to easily lyse, the sequences of the 16S rRNA genes targeted by the primers used to amplify the V1-V3 hypervariable regions from “*Ca*. M. girerdii” and *M. hominis* are identical to each other, and *M. hominis* strains typically have two 16S rRNA genes, in contrast to the single 16S rRNA gene in “*Ca*. M. girerdii,” these PCR-based data are consistent with a higher biomass of “*Ca*. M. girerdii” than of *M. hominis* in T. vaginalis-infected women. Consistent with the 16S rRNA survey of the vaginal microbiota, the proportions of mRNA-derived reads between the two *Mycoplasma* species in the *in vitro* cultures indicated a higher biomass for “*Ca*. M. girerdii” than for *M. hominis*. However, for all the native cultures of isolates with dual mycoplasma species, the MOI was inconsistent with this picture, with an opposite trend in their respective MOIs. This could be explained by the use of the TvSS-62–“*Ca*. M. girerdii” pair, the native symbiosis, where both partners may have adapted to each other. Variations observed between the *in vitro*- and *in vivo*-derived data could be due to variations in the concentrations of key metabolites in Diamond’s TYM medium and vaginal fluid/surfaces, respectively, with notably arginine being typically characterized by lower concentrations among BV patients (9). Such variations in metabolites could differentially impact the growth capabilities of “*Ca*. M. girerdii” versus *M. hominis* as arginine is known to represent the major source of energy for the latter but is unlikely to be relevant as an energy source for the former, which can use glycolysis based on genome annotation (27). The observed variation of MOIs for *M. hominis* measured by qPCR was consistent with previous data where T. vaginalis displays important isolate-to-isolate variability for *M. hominis* MOIs among clinical isolates as estimated by semiquantitative assays (47). All these data support the hypothesis that the capability of infection of “*Ca*. M. girerdii” may be inhibited by the presence of *M. hominis*, leading us to speculate on the existence of some form of competition between *M. hominis* and “*Ca.* M. girerdii” when in dual symbiosis with T. vaginalis. This hypothesis is also supported by our data obtained using our experimental infection model, which show that while “*Ca.* M. girerdii” can readily infect the axenic T. vaginalis recipient, the infection is much more difficult, if not impossible, when “*Ca.* M. girerdii” must infect T. vaginalis stably associated with *M. hominis* or when the two species of mycoplasma are coincubated with a mycoplasma-free parasite recipient. In the latter case, T. vaginalis establishes a stable relationship with *M. hominis* only, while “*Ca.* M. girerdii” is eliminated after a few days of cultivation. Moreover, we have observed the instability of the symbiosis between naturally mycoplasma-free T. vaginalis and “*Ca.* M. girerdii,” hypothesizing that such instability could be due to intrinsic difficulties of the naturally mycoplasma-free protist, potentially due to a lack of adaptation between the parasite and “*Ca.* M. girerdii.” These different considerations suggest complex metabolic interactions.

In order to evaluate the robustness of the “*Ca.* M. girerdii”-T. vaginalis association to some environmental changes, we performed starvation experiments with TvSS-62Mg, naturally in symbiosis with “*Ca.* M. girerdii,” and TvSS-62iso, experimentally cleaned from mycoplasmas. Upon short (30- and 60-min) starvation in PBS, the MOI of “*Ca.* M. girerdii” drastically decreases from ~12 bacteria per parasite in TvSS-62Mg to ~1 bacterium per trichomonad, indicating that unfavorable environmental conditions can strongly influence the ability of “*Ca*. M. girerdii” to infect the parasite or the parasite’s capacity to host the bacteria. These data were further supported by the results obtained by limiting dilution experiments assessed for TvSS-62Mg, where we detected genomic DNA of “*Ca*. M. girerdii” until a dilution of 12.5 T. vaginalis cells seeded per well in 100 μL of medium. This could be due to increased xenophagy of the bacteria by the parasite in response to this stress, as autophagy is known to be stimulated in T. vaginalis by glucose starvation (66). Alternatively, this could be due to the stress directly impacting the bacteria or a combination of these two processes.

The “*Ca.* M. girerdii”-T. vaginalis symbiosis is likely to be based on different metabolic interactions compared to the *M. hominis*-T. vaginalis consortium given the fundamentally different bases of energy metabolism identified for these two mycoplasma species: glycolysis in “*Ca.* M. girerdii” (27) and amino-acid-based (arginine) metabolism in *M. hominis* (24, 35). The T. vaginalis*-M. hominis* symbiosis brings together two ADH pathways exhibiting increased arginine consumption, concomitant with increases in ornithine and putrescine production (24). Notably, the addition of free arginine to culture medium is associated with an increase in the amount of ATP/cell in the T. vaginalis*-M. hominis* consortium, suggesting cross-beneficial metabolic interactions between the two symbiotic partners (22). Moreover, a recent study showed that under glucose restriction, T. vaginalis rapidly consumes arginine from the medium to generate ATP with a slight increase in proline levels (66). These findings support a model where the presence of *M. hominis* could help and promote the growth of T. vaginalis and could explain the higher MOI for *M. hominis* than for “*Ca*. M. girerdii” in T. vaginalis strains with double infection grown in TYM medium.

Another interesting finding was the demonstration of the intracellular localization of “*Ca.* M. girerdii” in T. vaginalis cells, given that previous fluorescence *in situ* hybridization (FISH) data rarely showed the bacteria colocalizing with T. vaginalis (27). Through a gentamicin protection assay, we found that the bacteria associated with T. vaginalis cells can survive under antibiotic exposure for 15 days. Notably, the number of bacteria associated with T. vaginalis cells was not statistically significantly different in parasites cultivated in the presence or absence of gentamicin (Table S7). Moreover, the intracellular location was further supported by fluorescence assays in T. vaginalis after cultivation in medium supplemented with gentamicin. The microscopy and qPCR data suggest that “*Ca.* M. girerdii” can live both on the cell surface and in an intracellular compartment in T. vaginalis cells, with a higher number of cells in the latter compartment. These data in combination thus suggest that the replication of “*Ca.* M. girerdii” in this system occurs mainly intracellularly, in contrast to *M. hominis*, which, even if it is able to multiply in the parasite cytoplasm, can also replicate, and substantially so, extracellularly under the tested *in vitro* growth conditions (45). These considerations are also consistent with the inability to grow “*Ca*. M. girerdii” *in vitro* despite numerous attempts (27).

10.1128/mbio.00918-22.10 TABLE S7 Number of “*Ca*. M. girerdii” bacteria associated with T. vaginalis growth in complete medium and medium with gentamicin added. Tv, T. vaginalis; Mg, “*Ca*. M. girerdii.” Download Table S7, DOCX file, 0.1 MB. Copyright © 2022 Margarita et al. 2022 Margarita et al. https://creativecommons.org/licenses/by/4.0/ This content is distributed under the terms of the Creative Commons Attribution 4.0 International license.

We have also performed comparative growth experiments to determine whether “*Ca*. M. girerdii” can influence parasite multiplication by studying the growth kinetics of T. vaginalis alone or coinfected with one or both mycoplasma species: T. vaginalis cultures associated with either mycoplasma or with both bacterial species promoted the parasite growth rate. The observed mycoplasma-dependent boost in T. vaginalis growth supports a model in which all three microbial species synergistically promote their respective survival and growth *in vivo*.

These results are also supported by the *in vitro* RNA-Seq analyses of T. vaginalis experimentally cleaned from “*Ca.* M. girerdii” (TvSS-62iso), T. vaginalis naturally associated with “*Ca.* M. girerdii” (TvSS-62Mg), T. vaginalis experimentally infected by *M. hominis* (TvSS-62iso+Mg), and doubly infected T. vaginalis (TvSS-62Mg+Mh). Consistent with the increase in the growth rate of T. vaginalis in symbiosis with *M. hominis* (22) and “*Ca*. M. girerdii,” RNA-Seq analyses indicated a major upregulation of T. vaginalis functions related to central energy metabolism and the storage of glycogen and a corresponding response to the potentially resultant increase in redox stress. Interestingly, the differential expression of central energy metabolism genes suggested a shift from hydrogenosomal metabolism toward cytosolic lactate and malate fermentation. Increased lactate dehydrogenase (LDH) expression may deplete pyruvate as a hydrogenosomal substrate, and hydrogenosomal malate consumption may be reduced by decreased malic enzyme expression (67). The significance of this shift is unclear, but synergistic *Trichomonas*-*Mycoplasma* metabolism could compensate for the loss of energy usually derived from substrate-level phosphorylation in the hydrogenosome. Alternatively, it was suggested previously by Westrop et al. (68) that some T. vaginalis LDH enzymes are involved in 2-hydroxy acid synthesis. As 2-hydroxy acids can inhibit microbial growth, it is possible that this response functions to limit *Mycoplasma* growth or to compete with other mucosal microorganisms *in vivo*. Increased amino acid catabolism may also have provided energy and biomass to support growth. The ADH pathways for energy generation shared by T. vaginalis and *M. hominis* suggest a potential synergistic metabolism. However, our transcriptional results concerning the ADH pathway are unclear. Overall, most enzymes showed little modulation in response to *Mycoplasma*. The influence of the opposite regulatory profiles of the 2 OCT homologs is difficult to interpret. Experimental characterization would be required to determine whether sequence differences between these proteins influence activity or posttranslational regulation. Interestingly, genes putatively annotated as arginase genes were upregulated in the presence of *Mycoplasma*. Previous evidence suggests that T. vaginalis cells lack arginase activity, although these experiments were likely conducted in the absence of both *M. hominis* and “*Ca.* M. girerdii” (69). Arginase could allow T. vaginalis to outcompete *M. hominis* for arginine to allow the continued synthesis of putrescine necessary for cell survival (68, 70, 71).

Ribosome biogenesis, a defined marker of an increased growth rate (72), was also increased in the presence of “*Ca*. M. girerdii” at the mRNA level.

The RNA compositional results between samples suggested an overall greater biomass of “*Ca.* M. girerdii” than of *M. hominis*, which may be consistent with the observed increase in expression at the mRNA level of lysosomal proteins specifically during symbiosis with *M. hominis*, which has also been observed in human cells infected with *M. hominis* (73). This may be involved in destroying some of the intracellular bacteria (45) through xenophagy, as was suggested previously by Vancini and Benchimol for T. vaginalis in symbiosis with *M. hominis* (44). These observations are congruent with a preference for the natural “*Ca*. M. girerdii” symbiont of the T. vaginalis strain (TvSS-62Mg) used for these experiments, as has been observed for other eukaryote-bacterium symbioses (74). In triple culture, “*Ca.* M. girerdii” appeared to outcompete *M. hominis* but also benefited from its presence in terms of its own abundance. Consistent with this, “*Ca.* M. girerdii” appeared to be the main driver of differential gene expression during the simultaneous symbiosis of T. vaginalis with *M. hominis*, as the expression profile under this condition most closely resembled that of the individual T. vaginalis-“*Ca.* M. girerdii” symbiosis and was distinct from that of T. vaginalis in symbiosis with *M. hominis*. Surprisingly, despite the major differences in the metabolic configurations between *M. hominis* and “*Ca.* M. girerdii,” there is a largely overlapping transcriptional response in T. vaginalis cells in symbiosis with these *Mycoplasma* species, including many common metabolic functions. We hypothesize that this may result from transcriptional regulation by *Trichomonas* in response to biochemical features common to both *Mycoplasma* species, such as their lipids.

*Mycoplasma* has been reported to influence various processes potentially related to T. vaginalis mucosal colonization and pathogenesis (22, 25, 75), which was reflected in our findings. The TvBspA-like gene family has been massively expanded, with over 900 members (37), substantial proportions of which were differentially expressed (255 genes) in response to the different symbioses with *Mycoplasma*. This potentially implicates the presence of *Mycoplasma* as a trigger to modulate host and T. vaginalis-microbe adhesion, particularly in the case of surface-localized TvBspA-like proteins verified by proteomics and other means (37, 54). Typically, LRR motifs are thought to facilitate protein-protein interactions (57), so TvBspA-like proteins may also play various roles in interactions with other cells, including host cells (53) and other microbes, such as the mycoplasmas themselves, and more generally could mediate binding to members of the microbiota that the parasite is known to bind to and phagocytose (76, 77). In support of this, we observed a complex regulatory pattern across the TvBspA-like gene family, with some genes being specifically upregulated in response to a single *Mycoplasma* species, suggesting a role in species-specific *Trichomonas*-*Mycoplasma* interactions. BspA-like genes were simultaneously expressed at a high level by “*Ca.* M. girerdii,” potentially providing a cognate interaction partner with the TvBspA-like protein, as demonstrated for BspA-like proteins from different species of oral bacteria (36).

In strong support of a host adhesion regulatory role of *Mycoplasma*, the majority, 8 of the 11 T. vaginalis genes, for which the corresponding proteins showed increased expression on the surface proteomes of more highly adherent T. vaginalis strains (54), were significantly upregulated at the mRNA level in response to the presence of *Mycoplasma*. Notably, higher levels of transcription of such genes were shown to be associated with higher levels of surface protein expression (54).

These data led us to investigate *in vitro* the influence of both mycoplasmas on two important aspects of T. vaginalis cytopathogenicity. In particular, we have assessed the ability of T. vaginalis-*Mycoplasma* consortia to lyse human RBCs compared with T. vaginalis alone: our data showed that both mycoplasma species were able to enhance the protozoan cytolytic effect, confirming our previous results on the impact of *M. hominis* endosymbiosis on the parasite hemolytic effect (22). We also evaluated the adherence capacity of T. vaginalis associated with one or both *Mycoplasma* species compared with *Mycoplasma*-free T. vaginalis on the basis of RNA-Seq results: our data demonstrate that the number of protist cells attached to epithelial cells was ~10-fold higher when symbiotically associated with one or both *Mycoplasma* species than with T. vaginalis alone.

A final consideration concerned oxidative stress tolerance, which is implicated in the virulence of various pathogens by providing resistance to ROS-mediated killing by immunocytes (78). Thus, the observed increase in ROS detoxification enzymes in response to *Mycoplasma* endosymbiosis could also be implicated in T. vaginalis pathobiology. This may work in synergy with another mechanism of tolerance mechanism to immunocyte-derived ROS thought to be facilitated by *M. hominis* via the catabolic depletion of arginine, the substrate for nitric oxide production (22).

This work highlights for the first time the stable intracellular relationship that “*Ca.* M. girerdii” forms with T. vaginalis and shows the ability of *M. hominis* to play a pivotal role in the relationships with the new mycoplasma species strictly associated with the parasite. The existence of such strongly intertwined microbial relationships in specific ecological niches in the human body depicts a picture of complex interactions between different microorganisms with pathogenic potential. Taken together, these findings support a model in which associations between T. vaginalis and vaginal mucosal bacteria are likely to influence and contribute to the broad diversity of the health sequelae associated with trichomoniasis. Future investigations should consider evaluating T. vaginalis-positive patients in combination with their *Mycoplasma* and TVV symbiosis status to determine whether such stratification could effectively predict a higher risk for preterm birth and/or HIV transmission/acquisition. Such patient stratifications would also have important implications for diagnostics, which could benefit from simultaneously detecting parasites, bacteria, and TVVs (21, 61).

## MATERIALS AND METHODS

### Analysis of published 16S rRNA data.

Previously published 16S rRNA data from mid-vaginal swab samples (27) were reanalyzed under study HM12169 as approved by the institutional review boards for human subject protection at the Virginia Commonwealth University and the Virginia Department of Health. Briefly, the V1-V3 hypervariable regions of the 16S rRNA gene were amplified using primers with a sequencing adaptor (shown in italics), a 6- to 9-base variable barcode sequence, and the 5′ end of the primer. The forward primer was a 4:1 mixture of primers Fwd-P1 (5′-*CCATCTCATCCCTGCGTGTCTCCGACTCAG*BBBBBBAGAGTTYGATYMTGGCTYAG) and Fwd-P2 (5′-*CCATCTCATCCCTGCGTGTCTCCGACTCA*GBBBBBBAGARTTTGATCYTGGTTCAG). The reverse primer was Rev1B (5′-CCTATCCCCTGTGTGCCTTGGCAGTCTCAGATTACCGCGGCTGCTGG).

PCR products were sequenced on the Roche 454 GS FLX Titanium platform. Sequencing reads with a valid primer and barcode were retained for analysis if they had fewer than 10% of base calls with a quality score of less than 10, an average quality score of greater than *Q*_20_, and a read length of between 200 and 540 bases. All analyzed samples had more than 5,000 reads. Taxonomic classification was performed using STIRRUPS (79). The analyzed data sets included profiles of 63 samples collected at the time of a trichomoniasis diagnosis and 73 women with detection of “*Ca.* M. girerdii” at 0.1% of the overall 16S rRNA profile. Among the 63 women with trichomoniasis, 28 samples had “*Ca.* M. girerdii” at 0.1%, 26 of which were available for inclusion in the group of 73 women with “*Ca.* M. girerdii.”

### Culture conditions for T. vaginalis and *Mycoplasma* species.

A total of 75 T. vaginalis strains previously isolated in the laboratory of microbiology of the University of Sassari from Italian female patients affected by acute trichomoniasis were investigated for the presence of *M. hominis* and “*Ca.* M. girerdii.” T. vaginalis strains were cultivated by daily passage at 1:16 in Diamond’s TYM (Trypticase, yeast extract, and maltose) medium supplemented with 10% fetal bovine serum (FBS) at 37°C in a 5% CO_2_ atmosphere (80) for 2 weeks. Genomic DNA was extracted with a commercial kit, the DNeasy blood and tissue kit (Qiagen Ltd., West Sussex, UK), according to the manufacturer’s protocols and analyzed by quantitative real-time PCR (qPCR).

A total of three T. vaginalis isolates were used for generating isogenic strains: T. vaginalis reference strain G3 (TvG3), naturally mycoplasma free; T. vaginalis strain SS-62 (TvSS-62Mg), naturally “*Ca.* M. girerdii” infected; and T. vaginalis strain SS-25 (TvSS-25MgMh), naturally associated with both *M. hominis* and “*Ca.* M. girerdii.”

*M. hominis* cells were isolated from T. vaginalis strain TvMPM2, which is naturally associated with *M. hominis* (47), and maintained in BEA medium (2.2% heart infusion broth, 15% horse serum, 1.9% yeast extract, 40 IU/mL benzylpenicillin, 0.23% l-arginine, 0.0023% phenol red [pH 7.2]) (81).

“*Ca*. M. girerdii” has not been isolated and maintained in culture in the absence of T. vaginalis despite several attempts to do so (27). In our experiments, we used bacteria from the supernatant of naturally “*Ca.* M. girerdii”-infected TvSS-62Mg cultures to experimentally infect mycoplasma-free and *M. hominis*-infected T. vaginalis strains.

### Quantitative real-time PCR assay for MOI determination.

qPCR assays for “*Ca.* M. girerdii” and *M. hominis* were performed using the CFX96 Touch real-time thermal cycler (Bio-Rad, Hercules, CA). Absolute quantification of “*Ca.* M. girerdii” and *M. hominis* DNA concentrations was performed by serial dilution of plasmids containing a single-copy housekeeping gene sequence. For “*Ca.* M. girerdii,” the full-length 16S rRNA gene cloned into pCR 2.1-TOPO was used as the standard. For *M. hominis*, a fully conserved gene fragment of a surface lipoprotein with nuclease activity (82), MHO_0730 (*M. hominis* strain ATCC 23114) cloned into pGEX2T, was used as the template to generate a standard curve. The primer sets used are listed in Table 5. The PCR mixture consisted of 2× SYBR select master mix (Applied Biosystems), 300 nM each primer, 10 to 100 ng/mL of the genomic DNA sample, and nuclease-free water (Invitrogen) to a volume of 20 μL.

**TABLE 5:** Primer sequences and amplicon sizes for *M. hominis* (Mh730 forward and reverse) and “*Ca.* M. girerdii” (OTU_M1 and OTU_M2)^*a*^

Primer	Sequence (5′–3′)	Amplicon size (bp)	Genome positions^*b*^
Mh730 forward	CCAAATCCTAAACCTGGTGGT	200	101490–101690
Mh730 reverse	CGGTTCACTCCAATTGCTTGAAAT		
OTU_M1 forward	CATTTCCTCTTAGTGCCGTTCG	310	408395–408790
OTU_M1 reverse	CGGAGGTAGCAATACCTTAGC		

a See reference 27.

b Relative positions in the Mycoplasma hominis reference strain A136 genome (GenBank accession number CP055143.1) and “*Candidatus* Mycoplasma girerdii” reference strain UC_B3 (GenBank accession number CP020122.1).

For “*Ca.* M. girerdii,” amplifications were performed for 2 min at 50°C and 10 min at 95°C, followed by 40 cycles of 15 s at 95°C and 1 min at 60°C. After the real-time PCR amplification was completed, a melting analysis was performed. The samples were heated to 95°C for 1 min, cooled to 60°C (0.10°C/s), and reheated to 95°C (0.5°C/s).

For *M. hominis*, the PCR program used was 2 min at 50°C and 10 min at 95°C, followed by 40 cycles with denaturation at 95°C for 15 s and annealing and elongation at 60°C for 1 min. After PCR, a melting program finalized the analysis.

Each sample was tested in triplicate, and negative and positive controls were processed in parallel in the same experiment.

### Gentamicin susceptibility of “*Ca.* M. girerdii” in symbiosis with T. vaginalis.

“*Ca.* M. girerdii”-infected TvSS-62Mg bacteria were cultivated in Diamond’s TYM medium supplemented with 50 μg mL^−1^ gentamicin. Aliquots of the culture were taken at different times (after 1, 3, 7, and 15 days of incubation with gentamicin) to assess long-term intracellular survival. Cells were centrifuged at 500 × *g* for 10 min; the cellular pellet was extensively washed in phosphate-buffered saline (PBS) three times and, together with the supernatant, was subjected to total DNA extraction as described above. Detection of “*Ca.* M. girerdii” DNA in the supernatant or associated with T. vaginalis whole cells was performed by qPCR. Furthermore, the presence of “*Ca.* M. girerdii” within trichomonad cells after 15 days of gentamicin treatment was investigated by fluorescence microscopy, staining the cells with 5 μg/mL DAPI (4′,6-diamidino-2-phenylindole) (see below). The supernatant from TvSS-62Mg cultures treated for 15 days was used to infect mycoplasma-free TvG3 in order to evaluate the residual ability to infect mycoplasma-free recipients. “*Ca*. M. girerdii”-infected TvSS-62Mg bacteria cultivated in Diamond’s TYM normal medium were used as controls.

### Experimental model to study the ability of *Mycoplasma* to infect T. vaginalis.

Isogenic T. vaginalis cultures were obtained by using two different approaches: (i) naturally mycoplasma-free TvG3 cells were infected with *M. hominis* or “*Ca.* M. girerdii” (24), or (ii) mycoplasma-infected strains TvSS-62Mg and TvSS-25MgMh were cleared of native mycoplasmas using Plasmocin (InvivoGen) as previously described (48). Mycoplasma-free T. vaginalis strains were subsequently used as the recipients and infected with the two *Mycoplasma* species in either single or double infections.

For the first approach, in order to obtain TvG3 stably infected with either *M. hominis* or “*Ca.* M. girerdii,” we collected the supernatants of TvMPM2Mh (naturally infected by *M. hominis*) or TvSS-62Mg (naturally infected by “*Ca.* M. girerdii”) by centrifuging cell cultures at 350 × *g* for 10 min. The supernatants containing 1.09E+05 *M. hominis* or 2.07E+06 “*Ca.* M. girerdii” cells, quantified based on qPCR, were filtered using a 0.22-μm-pore-size filter membrane and then separately added for 3 days to 10 mL of mid-log-phase TvG3 cells. To study the ability of *M. hominis* and “*Ca*. M. girerdii” to form a symbiosis with “*Ca*. M. girerdii”- or *M. hominis*-containing T. vaginalis, respectively, *M. hominis* in symbiosis with the TvG3 strain was incubated with “*Ca.* M. girerdii,” and “*Ca*. M. girerdii” in symbiosis with the TvG3 strain was incubated with *M. hominis* (Fig. 4A). Alternatively, *M. hominis* and “*Ca.* M. girerdii” were added at the same time to mycoplasma-free T. vaginalis cultures (Fig. 4D). Under all conditions, the different combinations of symbioses between mycoplasma and the parasite were cultivated for a further 15 days after exposure with 1:16 daily passages in Diamond’s TYM complete medium, and the ability of mycoplasma species to establish a stable single or double symbiosis was finally tested by qPCR to quantify the amount of mycoplasma cells associated with the parasites.

For the second approach, two different clinical isolates, TvSS-62Mg, naturally in symbiosis with “*Ca.* M. girerdii,” and TvSS-25MgMh, naturally in symbiosis with both “*Ca.* M. girerdii” and *M. hominis*, were treated to eliminate the endosymbiotic mycoplasmas through cultivation for 7 days in medium supplemented with Plasmocin (InvivoGen) at a final concentration of 25 μg/mL (48). Subsequently, cells were cultivated for 30 days in complete Diamond’s TYM medium to obtain isogenic mycoplasma-free T. vaginalis, named TvSS-62iso and TvSS-25iso, respectively. The absence of bacteria was confirmed by specific qPCRs, and the influence of treatment on the replication rate of the protist was assessed by comparing the growth curve of TvSS-62iso with that of TvG3, culturing the cells for 30 days. The axenic strains TvSS-25iso and TvSS-62iso were then used as the recipients for subsequent infections, superinfections, and coinfections by *M. hominis* and/or “*Ca.* M. girerdii,” as described above for the first approach. The variation of the MOI of *Mycoplasma* species in T. vaginalis during infection was evaluated by the extraction of DNA from aliquots of TvSS-62Mg and TvSS-62Mg+Mh cultures at 1, 7, 15, and 30 days of cultivation.

In order to avoid cross-contamination during cultivation, all isogenic parasite cultures were separately grown in different incubators and passaged daily using two different laminar flow hoods. The incubators and culture media were constantly monitored by qPCR to ensure that they were mycoplasma free.

### Fluorescence microscopy.

A volume of 1 mL of culture of TvSS-62Mg (naturally in symbiosis with “*Ca.* M. girerdii” and *M. hominis* free), TvSS-62iso (experimentally cleaned from “*Ca.* M. girerdii”), and TvSS-62Mg experimentally infected with *M. homini*s (TvSS-62Mg+Mh) was seeded into 24-well plates containing a round 12-mm-diameter coverslip in each well and incubated at 37°C in Diamond’s TYM medium for 24 h. The cells were then gently washed with PBS (pH 7.2), fixed with 4% paraformaldehyde in PBS for 1 h, and permeabilized in 2% Triton X-100 in PBS for 2 min. Cells were then stained with 5 μg/mL DAPI to detect “*Ca.* M. girerdii” and incubated for 1 h at 37°C with anti-*M. hominis* mouse polyclonal antibodies, obtained by inoculation of total mycoplasmal proteins into mice, with subsequent elution of sera tested to validate the specific reactivity to *M. hominis* and to exclude cross-reactivity with T. vaginalis, as described previously by Rappelli and colleagues (47). The cells were then incubated for 30 min at 37°C with tetramethyl rhodamine isothiocyanate (TRITC)-labeled goat anti-mouse antibodies (Sigma-Aldrich) to stain *M. hominis*.

The samples were observed using an Olympus BX51 fluorescence microscope, and the images were acquired with an Optronics MagnaFire charge-coupled-device (CCD) camera.

### *In vitro* multiplication rate of mycoplasma-infected T. vaginalis cultures and growth curve analyses.

The growth curves of four different isogenic associations, (i) mycoplasma-free T. vaginalis (TvSS-62iso), (ii) naturally “*Ca.* M. girerdii”-infected T. vaginalis (TvSS-62Mg), (iii) *M. hominis-*infected T. vaginalis (TvSS-62iso+Mh), and (iv) T. vaginalis infected by both mycoplasma species (TvSS-62Mg+Mh), were compared. Briefly, 400,000 parasite cells were inoculated into 10 mL of Diamond’s TYM medium and incubated at 37°C. Cell counts were recorded at 12, 18, 24, and 36 h postinoculation. The experiments were performed three times, each in triplicate.

The growth curves for the four different T. vaginalis isogenic conditions (TvSS-62Mg, TvSS-62iso, TvSS-62iso+Mh, and TvSS-62Mg+Mh) were fitted in R (Growthcurver) using the standard form of a logistic equation as a function of the growth rate (*r*), the initial population size (*N*_0_), and the carrying capacity (*K*). The equation to calculate the parasite population size (*N_t_*) at a given time (*t*) is

$$N_{t}=\frac{K}{1+\left(\frac{K-N_{0}}{N_{0}}\right)e^{-\mathrm{rt}}}$$

### Total RNA extraction.

TvSS-62Mg, naturally infected by “*Ca.* M. girerdii”; TvSS-62iso, experimentally cleaned from “*Ca.* M. girerdii”; TvSS-62Mh, experimentally infected by *M. hominis*; and T. vaginalis infected by “*Ca.* M. girerdii” and *M. hominis* (TvSS-62Mg+Mh) were collected in the exponential growth phase to a density of 2 × 10^6^ cells in a final volume of 40 mL of medium. Cells were centrifuged at 500 × *g* for 10 min, and cellular pellets were washed in 5 mL of PBS one time, resuspended in 700 μL of RNAlater (Thermo Fisher Scientific), and stored at −80°C. Material stored in RNAlater was thawed on ice, diluted with 0.7 mL nuclease-free PBS, and pelleted by centrifugation at 6,000 × *g* for 5 min at 4°C. RNA was extracted from the resulting pellet using TRIzol (Thermo Fisher Scientific) according to the manufacturer’s instructions, with some modifications. Briefly, after TRIzol and chloroform were used to lyse cells and solubilize cell components, RNA was precipitated from the aqueous phase, washed, and dissolved in 25 to 30 μL nuclease-free water. After washing the RNA pellet in 75% ethanol, centrifugation at 12,000 × *g* for 10 min at 4°C was used rather than the recommended 7,500 × *g* to improve the pelleting of the RNA.

### Quantification and quality analysis of RNA.

The RNA concentration was determined using the Qubit RNA high-sensitivity kit (Thermo Fisher Scientific) according to the manufacturer’s instructions. UV absorbance at 230 nm, 260 nm, and 280 nm was measured using a Nanodrop 2000c spectrophotometer as an indicator of purity. The absence of gDNA contamination and RNA integrity were confirmed using a TapeStation system (Agilent) by manually examining the resulting gel images and electropherograms, as the automatic RNA integrity number calculation (designed for eukaryotes) may not be suitable for mixed prokaryotic and eukaryotic total RNA.

### RNA sequencing.

All library preparation and Illumina sequencing were performed by Novogene UK. Libraries were prepared using a standard protocol, and prokaryotic and eukaryotic rRNAs were depleted using the Ribo-Zero kit (Illumina). Approximately 50 million paired-end reads per sample were generated using an Illumina NovaSeq 6000 platform. The read length was 150 bp, and the insert size was from 250 bp to 300 bp. Before obtaining the reads from Novogene UK, the adaptor sequences were removed, and sequences of low-quality reads (reads with >10% N’s [undetermined bases] or >50% of bases at or below a Phred quality score of 5) were deleted. Sequencing data are available from the NCBI SRA database (83) under accession numbers SRR12991837 to SRR12991843.

### *Trichomonas*-*Mycoplasma* coculture for RNA-Seq data analysis.

The workflow used to analyze gene expression from *Trichomonas*-*Mycoplasma* coculture RNA-Seq data is shown in Fig. S2 in the supplemental material. The average Phred quality score of all reads did not fall below 28 across the full read length as assessed by FastQC (84). Reads were filtered for rRNA sequences by alignment to a prokaryotic and eukaryotic rRNA database with SortMeRNA (49), Kraken2 was used to taxonomically assign sequences by a k-mer search against the NCBI nonredundant nucleotide database (50), and STAR was used to align reads to the reference genomes (85). SortMeRNA, Kraken2, and STAR were used with default parameters. The NCBI Taxonomy Toolkit (85) was used to agglomerate taxonomy identifications generated by Kraken2 to a specific taxonomic rank.

10.1128/mbio.00918-22.2 FIG S2 Flowchart describing the workflow used to analyze RNA-Seq data from *Trichomonas*-*Mycoplasma* coculture. (1) Read quality assessment by FastQC (S. W. Wingett and S. Andrews, F1000Res 7:1338, 2018, https://doi.org/10.12688/f1000research.15931.2). (2) Alignment to prokaryotic and eukaryotic rRNA databases with SortMeRNA (E. Kopylova, L. Noé, and H. Touzet, Bioinformatics 28:3211–3217, 2012, https://doi.org/10.1093/bioinformatics/bts611). (3) k-mer search of the NCBI nonredundant nucleotide database (86) with Kraken2 (D. E. Wood, J. Lu, and B. Langmead, Genome Biol 20:257, 2019, https://doi.org/10.1186/s13059-019-1891-0). (4) Read alignment to the reference genome with STAR (84). (5) Test for differential gene expression using the R package edgeR (D. J. McCarthy, Y. Chen, and G. K. Smyth, Nucleic Acids Res 40:4288–4297, 2012, https://doi.org/10.1093/nar/gks042). (6) GO function enrichment analysis with PANTHER (93). (7) KEGG pathway enrichment analysis with edgeR (96; McCarthy et al., Nucleic Acids Res 40:4288–4297, 2012, https://doi.org/10.1093/nar/gks042). Download FIG S2, DOCX file, 0.1 MB. Copyright © 2022 Margarita et al. 2022 Margarita et al. https://creativecommons.org/licenses/by/4.0/ This content is distributed under the terms of the Creative Commons Attribution 4.0 International license.

The T. vaginalis G3 genome (NCBI accession number ASM282v1) (46) was used as the parasite reference sequence, and additional annotation information was obtained for BspA-like genes from Noël and colleagues (37), for experimentally verified surface proteins (EVSPs) from the study of de Miguel and colleagues (54), and for exosomes from Twu and colleagues (55). To select the best *Mycoplasma* reference genome, *Mycoplasma* reads were aligned to available genomes from the NCBI for the corresponding species (86). *M. hominis* accession number ASM93586v1 (strain PL5) and “*Ca.* M. girerdii” accession number ASM221542v1 (strain UC_B3) were selected as showing the best alignment statistics. A decision matrix was used to compare alignment metrics for the larger number of *M. hominis* reference sequences. The overall score was calculated by multiplying the average alignment length, the percent base mismatch rate, the percentage of reads for which the alignment length was too short, the percentage of reads that did not align for other reasons, and the percentage of reads that aligned uniquely, taking the reciprocal where lower values were better, scaling values between 1 and 5, and multiplying by weights of 1, 5, 5, 10, and 10 for each metric, respectively. Genes with an expression level of at least 1 transcript per million (TPM) were considered to be expressed.

SAMtools (87) was used to manipulate alignment files. To assess sequence differences between the reference and experimental strains for genes of interest, BCftools (88) and VCFutils (89) were used to generate a consensus sequence based on the most frequent sequence variants. A *de novo* assembly of reads assigned as unclassified (ranging from 14 to 27% of the total reads) by Kraken2 was generated using rnaSPAdes (90) to investigate their identity. Reads classified as “other” (ranging from 4.3 to 8.2% of the total reads) aligned to species that were not expected to be present within the experiment. Figure S3 shows that the large majority of the assembled transcripts were short, and the mean transcript length ranged from 400 to 450 bp, which is only slightly longer than the paired-read length (300 bp).

10.1128/mbio.00918-22.3 FIG S3 Kernel density plot showing the distribution of transcript lengths assembled from “unclassified” *Trichomonas-Mycoplasma* coculture reads by SPAdes. The vertical black line is shown at 300 bp. Download FIG S3, DOCX file, 0.1 MB. Copyright © 2022 Margarita et al. 2022 Margarita et al. https://creativecommons.org/licenses/by/4.0/ This content is distributed under the terms of the Creative Commons Attribution 4.0 International license.

The edgeR R package (91) was used to test for the differential gene expression of T. vaginalis annotated genes (46) using the negative binomial generalized linear model with a quasilikelihood test, considering only genes with a log_2_ fold change of greater than 1.2 for testing. Expression levels are presented as either the trimmed mean of M values (TMM) (91) or TPM, the latter calculated by the following equation:

$$\text{TPM}=\frac{\text{mapped reads/transcript length}}{\sum^{\;}(\text{mapped reads/transcript length})}\times 10^{6}$$

The TPM or z-scaled TMM was used to generate heat maps according to the range of expression of a given gene set (92).

For gene ontology (GO) enrichment analysis, PANTHER (93) was used, the full set of T. vaginalis genes detected to be expressed in the experiment was used as a reference database, and uninformative and redundant enriched functions were removed manually. KEGG enrichment analysis was performed using edgeR (92). For significance tests of differential gene expression and functionally enriched KEGG pathways and GO functions, *P* values were adjusted using the false discovery rate/Benjamini-Hochberg (FDR/BH) method. Testing for the differential expression of genes as a set was performed using the rotation gene set test (94) in edgeR.

A DiVenn (95) figure was created to depict the overlap of differentially expressed genes by T. vaginalis between the tested conditions.

KEGG metabolic pathways were predicted for “*Ca*. M. girerdii” and *M. hominis* using the online BlastKoala tool (96), using default parameters and the default reference database of prokaryotic genomes with redundancy removed at the genus level. T. vaginalis metabolic pathways were retrieved from the KEGG database (96).

The structural organization and cellular localization of specific proteins of interest were predicted using InterProScan (97).

### Variability of “*Ca.* M. girerdii” MOIs in TvSS-62Mg under stress conditions.

TvSS-62Mg, naturally “*Ca.* M. girerdii” infected, and TvSS-62iso (experimentally cleaned from “*Ca.* M. girerdii”) were grown for 30 min and 60 min in PBS–1 M maltose medium, thus depriving microorganisms of a broad range of nutrients, and then cultivated for a further 24 h in complete Diamond’s TYM medium. Following the starvation period, the “*Ca.* M. girerdii” MOI was calculated by using qPCR as described above. TvSS-62Mg and TvSS-62iso, normally grown for 24 h in Diamond’s TYM medium and not starved, were used as controls.

Moreover, TvSS-62Mg underwent limiting dilution from 1 × 10^2^ cells to 1 cell/well in complete Diamond’s TYM medium and incubated in 96-well plates under anaerobic conditions for 10 days. DNA extraction and qPCR were performed to evaluate the presence of “*Ca.* M. girerdii” in all dilutions.

### Hemolytic activity of *Mycoplasma*-infected strains.

T. vaginalis SS-62Mg, associated with “*Ca.* M. girerdii,” and T. vaginalis SS-62Mg+Mh, associated with both mycoplasmas, were compared with isogenic T. vaginalis SS-62iso, experimentally mycoplasma free, to evaluate hemolytic activity. Hemolysis assays were performed as previously described (60). Briefly, RBCs were collected from healthy human donors; erythrocytes were then washed three times in PBS and immediately used. Parasites in the exponential growth phase were washed twice in PBS and resuspended to a density of 2 × 10^6^ cells in PBS plus 15 mM maltose (PBS-M). T. vaginalis isogenic strains were incubated at 37°C with washed erythrocytes at a ratio of 1:30 in PBS-M. The hemoglobin released after incubation with RBCs for different times ranging from 90 to 180 min was evaluated by spectrophotometric analysis at a 546-nm absorbance. The hemolytic capacity of “*Ca.* M. girerdii”-infected T. vaginalis and the doubly infected protist was compared to that of parental uninfected isogenic T. vaginalis SS-62iso.

### Adherence assay of T. vaginalis isogenic strains.

A T. vaginalis binding assay was carried out with a modified version of a method described previously (98). Briefly, oral keratinocytes (NOK) (99) and HeLa cells (ATCC CCL-2) were seeded into 24-well plates at 1.75 × 10^5^ cells/well in culture medium and grown to confluence at 37°C with 5% CO_2_ for 2 days. T. vaginalis isogenic strains (TvSS-62iso, TvSS-62Mg, TvSS-62Mh, and TvSS-62Mg+Mh) were added at a concentration of 10^5^ cells/mL to NOK cells in triplicate. Plates were incubated at 37°C in 5% CO_2_ for 30 min, and the monolayers were washed 2 times in PBS to remove unbound parasites. Subsequently, the cells (human and parasite cells with or without mycoplasma) were detached using trypsin, and DNA extraction was performed using the DNeasy blood and tissue kit. The amount of T. vaginalis cells bound to NOK cells was analyzed by qPCR, using actin (TVAG_534990) as a gene target.

### Statistical analysis.

All experiments were carried out at least in triplicate. Statistical analyses were conducted in R (100) and Microsoft Excel (Microsoft, Redmond, WA, USA) with the indicated tests. A *P* value of <0.05 was considered significant.
