# Supplementary material for: Two Different Species of Mycoplasma Endosymbionts Can Influence Trichomonas vaginalis Pathophysiology
Source: mBio. 2022 May 24;13(3):e00918-22. doi: 10.1128/mbio.00918-22 (PMC9239101; doi:10.1128/mbio.00918-22)
Supplement: TABLE S1 [file mbio.00918-22-s0003.docx]

**S1 Table. 16SrRNA profiling data from the 73 women with ‘*Ca*. M. girerdii’**.

|  | 16S rRNA microbiome profiles of women with a proportional abundance of at least 0.1% ‘*Ca*. M. girerdii’N=73 |
| --- | --- |
| Tv+/*M. hominis* + | 49% (36/73) |
| Tv+/*M. hominis* - | 34% (25/73) |
| Tv-/M. hominis + | 4% (3/73) |
| Tv-/M. hominis - | 11% (8/73) |
| Tv ND/ M. hominis + | 1% (1/73) |

Tv, *T. vaginalis*; Tv ND, *T. vaginalis* no date; +, positive, - negative.

A threshold of 0.1% of total 16S rRNA reads was used as the threshold for presence of ‘*Ca*. M. girerdii’ and *M. hominis*.
