# Supplementary material for: Two Different Species of Mycoplasma Endosymbionts Can Influence Trichomonas vaginalis Pathophysiology
Source: mBio. 2022 May 24;13(3):e00918-22. doi: 10.1128/mbio.00918-22 (PMC9239101; doi:10.1128/mbio.00918-22)
Supplement: TABLE S2 [file mbio.00918-22-s0004.docx]

**S2 Table. Number of *Mycoplasma* species associated with 22 different *T. vaginalis* isolates.**

| Strain code | Tv cells concentration | Number of ‘*Ca*.M.girerdii’ | MOI Mg:Tv | MOI Mh:Tv | Number of *M.hominis* |
| --- | --- | --- | --- | --- | --- |
| SS-01 | 1.20E+06 | 2.41E+04  2.39E+04  1.50E+04 | 0.02  0.02  0.012 | 8.68  8.40  7.57 | 1.04E+07  1.01E+07  9.09E+06 |
| SS-02 | 8.00E+05 | 1.68E+04  1.44E+04  8.68E+03 | 0.02  0.02  0.01 | 0.002  0.0002  0.0002 | 1.63E+03  1.27E+02  1.74E+02 |
| SS-03 | 1.00E+06 | 2.97E+05  3.68E+05  1.76E+04 | 0.30  0.37  0.02 | 1.53  2.06  1.99 | 1.53E+06  2.06E+06  1.99E+06 |
| SS-09 | 1.20E+06 | 2.75E+05  2.36E+05  1.61E+04 | 0.23  0.20  0.01 | 11.22  19.63  8.72 | 1.35E+07  2.36E+07  1.05E+07 |
| SS-10 | 1.00E+06 | 3.11E+04  2.31E+04  2.49E+04 | 0.03  0.02  0.02 | 10.28  10.35  10.75 | 1.03E+07  1.03E+07  1.07E+07 |
| SS-17 | 1.30E+06 | 2.31E+04  2.49E+04 | 0.02  0.02 | 63.90  54.07 | 8.31E+07  7.03E+07 |
| SS-20 | 8.00E+05 | 2.29E+04  2.26E+04  1.91E+04 | 0.03  0.03  0.02 | 55.58  62.42  1.71 | 4.45E+07  4.99E+07  1.37E+06 |
| SS-25 | 8.00E+05 | 2.45E+04  9.64E+02  6.96E+02 | 0.03  0.001  0.001 | 0.21  0.65  0.17 | 1.67E+05  5.21E+05  1.34E+05 |
| SS-31 | 8.00E+05 | 1.36E+05  6.51E+04 | 0.2  0.1 | 2.95  1.83 | 2.36E+06  1.46E+06 |
| SS-35 | 1.50E+06 | 2.72E+05  3.71E+05  3.46E+04 | 0.18  0.25  0.02 | 49.25  41.95  40.13 | 7.39E+07  6.29E+07  6.02E+07 |
| SS-41 | 1.20E+06 | 2.98E+04  2.98E+04  2.06E+04 | 0.02  0.02  0.02 | 151.63  159.04  133.96 | 1.82E+08  1.91E+08  1.61E+08 |
| SS-42 | 8.00E+05 | 1.96E+04  3.02E+04  9.17E+03 | 0.025  0.038  0.011 | 1.49  2.60  1.38 | 1.19E+06  2.08E+06  1.10E+06 |
| SS-45 | 1.30E+06 | 2.74E+05  2.53E+05  8.27E+04 | 0.21  0.19  0.06 | 18.22  21.94  18.01 | 2.37E+07  2.85E+07  2.34E+07 |
| SS-60 | 1.00E+06 | 2.78E+04  2.03E+04 | 0.03  0.02 | 1.55  1.49 | 1.55E+06  1.49E+06 |
| SS-77 | 4.00E+05 | 1.60E+04  1.66E+04  9.97E+03 | 0.04  0.04  0.02 | 23.22  27.36  33.83 | 9.29E+06  1.09E+07  1.35E+07 |
| SS-78 | 1.00E+06 | 3.45E+03  4.68E+03  1.21E+03 | 0.003  0.005  0.001 | 0.07  0.06  0.06 | 3.26E+04  3.17E+04  3.15E+04 |
| SS-84 | 1.00E+06 | 4.10E+03  4.24E+03  3.62E+03 | 0.004  0.004  0.004 | 1.7  1.7  1.5 | 1.71E+06  1.68E+06  1.52E+06 |
| SS-88 | 1.30E+06 | 1.82E+04  2.26E+04  3.99E+02 | 0.01  0.02  0.0003 | 17.35  19.14  19.75 | 2.26E+07  2.49E+07  2.57E+07 |
| SS-90 | 1.00E+06 | 2.25E+04  4.99E+04 | 0.02  0.05 | 120  20 | 1.20E+08  1.98E+07 |
| TO-1 | 8.00E+05 | 3.55E+04  3.55E+04  3.33E+04 | 0.04  0.04  0.04 | 16.2  16.2  65.0 | 1.29E+07  1.29E+07  5.20E+07 |
| TO-3 | 1.20E+06 | 2.39E+05  2.17E+05  8.17E+03 | 0.20  0.18  0.01 | 43  63  57 | 5.20E+07  7.52E+07  6.84E+07 |
| MO-4 | 1.00E+06 | 3.46E+03  2.55E+03  2.88E+03 | 0.003  0.003  0.003 | 2.9  2.3  3.6 | 1.47E+06  1.15E+06  1.80E+06 |
| MO-5 | 1.00E+06 | 1.14E+04  1.03E+04  1.44E+04 | 0.011  0.010  0.014 | 38.2  44.8  40.2 | 3.82E+07  4.48E+07  4.02E+07 |
| MO-6 | 5.00E+05 | 3.07E+03  4.24E+03  3.49E+03 | 0.006  0.008  0.007 | 1.7  2.3  1.9 | 8.47E+05  1.16E+06  9.61E+05 |
| MO-7 | 7.00E+05 | 2.45E+03  1.83E+03  1.57E+03 | 0.004  0.003  0.002 | 1.1  1.0  1.0 | 7.85E+05  7.19E+05  6.90E+05 |
| NU-02 | 1.20E+06 | 2.40E+04  1.13E+04  1.21E+04 | 0.020  0.009  0.010 | 5.4  5.2  4.2 | 6.43E+06  6.29E+06  5.00E+06 |
| NU-03 | 1.90E+06 | 4.61E+04  4.45E+04  4.86E+04 | 0.024  0.023  0.026 | 44.1  68.2  49.5 | 8.39E+07  1.30E+08  9.40E+07 |
| NU-04 | 8.00E+05 | 2.14E+04  2.86E+03  1.29E+04 | 0.027  0.004  0.016 | 4.6  17.4  21.9 | 3.65E+06  1.39E+07  1.75E+07 |
| NU-05 | 6.50E+05 | 1.12E+04  1.38E+04  8.98E+03 | 0.017  0.021  0.014 | 13.9  21.6  17.3 | 9.01E+06  1.40E+07  1.13E+07 |
| NU-06 | 4.00E+05 | 1.38E+03  1.46E+03  9.85E+02 | 0.003  0.002  0.001 | 1.2  1.1  1.1 | 4.62E+05  4.30E+05  4.57E+05 |
| TO-5 | 1.50E+06 | 4.04E+04  4.16E+04  4.12E+04 | 0.027  0.028  0.027 | 8.8  15.8  12.4 | 1.32E+07  2.38E+07  1.86E+07 |
| TO-6 | 8.00E+05 | 2.10E+04  7.56E+03  7.45E+03 | 0.03  0.01  0.01 | 3.4  3.5  3.7 | 2.71E+06  2.83E+06  2.98E+06 |
| TO-7 | 1.20E+06 | 7.74E+05  3.00E+05  5.92E+05 | 0.64  0.25  0.49 | 1.2  1.1  1.2 | 1.38E+06  1.37E+06  1.40E+06 |
| TO-8 | 1.20E+06 | 8.82E+04  6.71E+04  7.40E+04 | 0.07  0.06  0.06 | 8.2  8.9  8.0 | 9.79E+06  1.07E+07  9.57E+06 |
| TO-9 | 5.00E+05 | 8.18E+03  5.95E+04  1.09E+04 | 0.02  0.12  0.02 | 4.2  4.7  4.5 | 2.11E+06  2.36E+06  2.25E+06 |
| TO-10 | 1.00E+06 | 5.50E+03  2.01E+04 | 0.01  0.02 | 3.7  1.5 | 3.65E+06  1.46E+06 |
| TO-11 | 1.00E+06 | 5.03E+05  1.11E+05  1.38E+05 | 0.50  0.11  0.14 | 23.8  9.8  11.3 | 2.38E+07  9.83E+06  1.13E+07 |
| SS-95 | 8.00E+05 | 5.03E+04  6.29E+02  1.34E+03 | 0.05  0.001  0.001 | 0.4  0.4  0.5 | 3.37E+05  3.35E+05  4.02E+05 |
| SS-96 | 1.50E+06 | 1.16E+04  5.03E+04  9.40E+03 | 0.01  0.05  0.01 | 43.9  33.5  30.0 | 6.58E+07  5.03E+07  4.50E+07 |
| SS-69 | 1.20E+06 | 2.79E+05  1.97E+05  1.46E+05 | 0.28  0.20  0.15 | 20.97  26.11  22.70 | 2.52E+07  3.13E+07  2.72E+07 |
| SS-70 | 5.00E+05 | 8.11E+03  5.00E+03  5.11E+03 | 0.02  0.01  0.01 | 7.74  7.41  8.51 | 3.87E+06  3.70E+06  4.26E+06 |
| SS-U2 | 8.00E+05 | 1.09E+05  6.89E+04  2.10E+05 | 0.14  0.09  0.26 | 17.53  20.56  20.97 | 1.40E+07  1.65E+07  1.68E+07 |

Tv, *T. vaginalis*; Mg, ‘*Ca.* M. girerdii’; Mh, *M. hominis*
