# Supplementary material for: Two Different Species of Mycoplasma Endosymbionts Can Influence Trichomonas vaginalis Pathophysiology
Source: mBio. 2022 May 24;13(3):e00918-22. doi: 10.1128/mbio.00918-22 (PMC9239101; doi:10.1128/mbio.00918-22)
Supplement: FIG S1 [file mbio.00918-22-s0010.docx]

**Supplementary Figure 1**

**Dynamics of infection TvSS62 isogenic strains.** (A) *T. vaginalis* experimentally cleaned by ‘*Ca*. M. girerdii’ and *T. vaginalis* G3, naturally *Mycoplasma* free, were cultivated for 30 days in order to assess the influence of Plasmocin treatment of growth of protist. (B) The number of ‘*Ca*. M. girerdii’ was compared between *T. vaginalis* SS-62, naturally ‘*Ca*. M. girerdii’ infected, and TvSS62Mg experimentally infected with *M. hominis* and, as shown in the graph, the number of ‘*Ca*. M. girerdii’ statistically decrease when *M. hominis* infection is stabilised in trichomonad cells (p<0.01)
