# Supplementary material for: Two Different Species of Mycoplasma Endosymbionts Can Influence Trichomonas vaginalis Pathophysiology
Source: mBio. 2022 May 24;13(3):e00918-22. doi: 10.1128/mbio.00918-22 (PMC9239101; doi:10.1128/mbio.00918-22)
Supplement: TABLE S3 [file mbio.00918-22-s0005.docx]

Supplementary Table 3

Because the file is too large (more than 10 MB), you have utilized a link to “*fighshare*”

<https://figshare.com/s/dbd42153e61a46803187>
