# Supplementary material for: Two Different Species of Mycoplasma Endosymbionts Can Influence Trichomonas vaginalis Pathophysiology
Source: mBio. 2022 May 24;13(3):e00918-22. doi: 10.1128/mbio.00918-22 (PMC9239101; doi:10.1128/mbio.00918-22)
Supplement: TABLE S7 [file mbio.00918-22-s0009.docx]

**S7 Table. Number of ‘*Ca*. M. girerdii’ associated with *T. vaginalis* growth in complete medium and in medium added with gentamicin**

|  | 1d of cultivation | | 15d of cultivation | |
| --- | --- | --- | --- | --- |
|  | **Tv cells concentration** | Number of Mg associate with protist | **Tv cells concentration** | **Number of Mg associate with protist** |
| TvSS-62Mg | 1.20E+06 | \| 8.65E+07 \| \| --- \| \| 9.14E+07 \| \| 3.32E+06 \| | 1.00E+06 | \| 5.25E+06 \| \| --- \| \| 8.67E+07 \| \| 4.77E+07 \| |
| TvSS-62Mg + gentamycin | 7.50E+05 | \| 3.92E+06 \| \| --- \| \| 1.68E+07 \| \| 2.15E+07 \| | 1.14E+06 | \| 1.39E+06 \| \| --- \| \| 5.17E+06 \| \| 4.93E+06 \| |

Tv, *T. vaginalis*; Mg, ‘*Ca*. M. girerdii’
