# Supplementary material for: Two Different Species of Mycoplasma Endosymbionts Can Influence Trichomonas vaginalis Pathophysiology
Source: mBio. 2022 May 24;13(3):e00918-22. doi: 10.1128/mbio.00918-22 (PMC9239101; doi:10.1128/mbio.00918-22)
Supplement: FIG S2 [file mbio.00918-22-s0001.docx]

**Supplementary Figure 2.**

**Flow chart describing the workflow used to analyse RNA-Seq data from *Trichomonas*-*Mycoplasma* co-culture**. 1. Read quality assessment with FastQC (101), 2. Alignment to prokaryotic and eukaryotic rRNA database with SortMeRNA (102), 3. k-mer search of NCBI non-redundant nucleotide database (86) with Kraken2 (103), 4. Read alignment to reference genome with STAR (84), 5. Test for differential gene expression using the R package edgeR (104), 6. GO function enrichment analysis with PANTHER (93), 7. KEGG pathway enrichment analysis with edgeR (96, 104).

Supplementary References

101. Wingett SW, Andrews S. 2018. FastQ Screen: A tool for multi-genome mapping and quality control. F1000Research 7:1338.

102. Kopylova E, Noé L, Touzet H. 2012. SortMeRNA: fast and accurate filtering of ribosomal RNAs in metatranscriptomic data. Bioinformatics 28:3211–3217.

103. Wood DE, Lu J, Langmead B. 2019. Improved metagenomic analysis with Kraken 2. Genome Biol 20:257.

104. McCarthy DJ, Chen Y, Smyth GK. 2012. Differential expression analysis of multifactor RNA-Seq experiments with respect to biological variation. Nucleic Acids Res 40:4288-4297.
