# Supplementary material for: Two Different Species of Mycoplasma Endosymbionts Can Influence Trichomonas vaginalis Pathophysiology
Source: mBio. 2022 May 24;13(3):e00918-22. doi: 10.1128/mbio.00918-22 (PMC9239101; doi:10.1128/mbio.00918-22)
Supplement: FIG S3 [file mbio.00918-22-s0002.docx]

**Supplementary Figure 3**

**
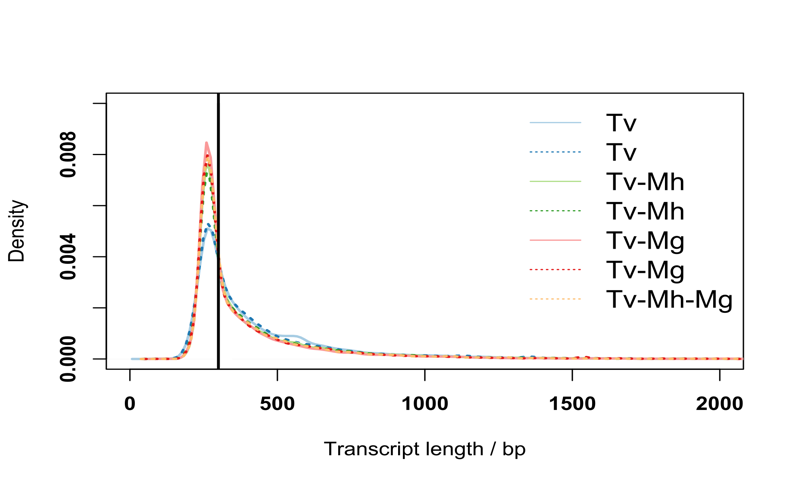
**

**Kernel density plot showing the distribution of transcript lengths assembled from “unclassified” Trichomonas-Mycoplasma co-culture reads by SPAdes.** Vertical black line is shown at 300 bp.
